# Sputnik V Effectiveness against Hospitalization with COVID-19 during Omicron Dominance

**DOI:** 10.3390/vaccines10060938

**Published:** 2022-06-13

**Authors:** Andrey S. Shkoda, Vladimir A. Gushchin, Darya A. Ogarkova, Svetlana V. Stavitskaya, Olga E. Orlova, Nadezhda A. Kuznetsova, Elena N. Keruntu, Andrei A. Pochtovyi, Alexander V. Pukhov, Denis A. Kleymenov, Vasyli G. Krzhanovsky, Daria V. Vasina, Nataliya V. Shkuratova, Elena V. Shidlovskaya, Alexey L. Gorbunov, Daria D. Kustova, Evgeniya A. Mazurina, Sofya R. Kozlova, Alexandra V. Soboleva, Igor V. Grigoriev, Lyudmila L. Pankratyeva, Alina S. Odintsova, Elizaveta D. Belyaeva, Arina A. Bessonova, Lyudmila A. Vasilchenko, Igor P. Lupu, Ruslan R. Adgamov, Artem P. Tkachuk, Elizaveta A. Tokarskaya, Denis Y. Logunov, Alexander L. Gintsburg

**Affiliations:** 1 City Clinical Hospital No. 67 Named after. L.A. Vorokhobov of the Moscow City Department, 123423 Moscow, Russia; a.shkoda@67gkb.ru (A.S.S.); s.stavitskaya@67gkb.ru (S.V.S.); o.orlova@67gkb.ru (O.E.O.); ekeruntu@yandex.ru (E.N.K.); a.puhov@67gkb.ru (A.V.P.); kryzhanv@ya.ru (V.G.K.); 1-rao@67gkb.ru (N.V.S.); 4-ho@67gkb.ru (A.L.G.); e.mazurina@67gkb.ru (E.A.M.); a.soboleva@67gkb.ru (A.V.S.); liudmila.pankratyeva@gmail.com (L.L.P.); 2 Federal State Budget Institution “National Research Centre for Epidemiology and Microbiology Named after Honorary Academician N F Gamaleya” of the Ministry of Health of the Russian Federation, 123098 Moscow, Russia; DashaDv1993@gmail.com (D.A.O.); nadyakuznetsova0@gmail.com (N.A.K.); a.pochtovyy@gmail.com (A.A.P.); mne10000let@gmail.com (D.A.K.); d.v.vasina@gmail.com (D.V.V.); lenitsa@gmail.com (E.V.S.); kustovad70@gmail.com (D.D.K.); sofya_dadashyan@mail.ru (S.R.K.); iggrigoriev.ltb@gmail.com (I.V.G.); avocnido.anila@gmail.com (A.S.O.); belyaevaliza2000@gmail.com (E.D.B.); abessonova1705@gmail.com (A.A.B.); milavasilchenko97@gmail.com (L.A.V.); vlad.cep888@yandex.ru (I.P.L.); bacter@yandex.ru (R.R.A.); artem.p.tkachuk@gmail.com (A.P.T.); liza18068q@ya.ru (E.A.T.); gintsburg@gamaleya.org (A.L.G.); 3 Department of Virology, Biological Faculty, Lomonosov Moscow State University, 119991 Moscow, Russia; 4 Department of Infectiology and Virology, Federal State Autonomous Educational Institution of Higher Education I M Sechenov First Moscow State Medical University of the Ministry of Health of the Russian Federation (Sechenov University), 119435 Moscow, Russia

**Keywords:** COVID-19, SARS-CoV-2, vaccine, Sputnik V, Sputnik Light, VOC, Omicron, vaccine effectiveness

## Abstract

Mass vaccination campaigns against COVID-19 affected more than 90% of the population in most developed countries. The new epidemiologic wave of COVID-19 has been ongoing since the end of 2021. It is caused by a virus variant B.1.1.529, also known as “Omicron” and its descendants. The effectiveness of major vaccines against Omicron is not known. The purpose of this study is to evaluate the efficacy of the Sputnik V vaccine. The main goal is to assess its protection against hospitalization in the period of Omicron dominance. We conducted our study based on a large clinical center in Moscow (Russia) where 1112 patients were included. We used the case-population method to perform the calculations. The data we obtained indicate that the Omicron variant causes at least 90% of infections in the studied cohort. The effectiveness of protection against hospitalization with COVID-19 in our study was 85.9% (95% CI 83.0–88.0%) for those who received more than one dose. It was 87.6% (95% CI 85.4–89.5%) and 97.0% (95% CI 95.9–97.8%) for those who received more than two or three doses. The effectiveness in cases of more severe forms was higher than for less severe ones. Thus, present study indicates the high protective efficacy of vaccination against hospitalization with COVID-19 in case of Omicron lineage.

## 1. Introduction

The emergence of several vaccines with high efficacy for COVID-19 prevention was the greatest success of the first pandemic year, including Sputnik V (Gamaleya Research Centre) [1], mRNA-1273 (Moderna) [2], BNT162b2 (Pfizer) [3], and ChAdOx1 nCoV-19 Vaccine AZD1222 (Oxford, UK, AstraZeneca) [4]. The efficacy of adenoviral vector and mRNA platforms was rather high. It exceeded 90% concerning protection against the disease and 95% concerning protection against hospitalization, according to randomized double-blind clinical studies. This prompted the launch of extensive vaccination programs around the world [5]. According to the field studies, the achievement of high vaccination levels in several developed [6] and developing [7,8] countries has demonstrated the high effectiveness of such vaccines. What is more, it has significantly reduced the incidence and need for the hospitalization of patients with COVID-19 for some time [9].

Unfortunately, new variants of concern (VOC) began to appear (including Alpha B.1.1.7, Beta B.1.351, Gamma P.1, Delta B.1.1617.2, and Omicron B.1.1.529) [10]. All VOC had mutations in the receptor-binding domain (RBD) of the S protein, as well as many mutations in other parts of the genome. This led to increased transmissivity and the ability to partially escape from the virus-neutralizing activity of antibodies gained following vaccination and previous disease.

Omicron appeared to be the champion in the number of new mutations [11]. In total, this variant has more than 50 mutations, 17 of which are in the RBD region. The wide distribution of the Omicron variant has led to the largest wave of COVID-19 morbidity in the world. The increase in cases has been recorded since November 2021 [12]. In April 2022, this wave is decreasing. To note, the largest wave of Omicron incidence occurred amid a high percentage of vaccinations in developed countries. Booster immunization (re-vaccination) had already been widely used in many countries during the emergence of Omicron. Re-vaccination occurred 5–6 months following the initial course of two injections. The recommendation for booster immunization was developed during the period of the Delta variant distribution [13,14].

The wide distribution of the Omicron variant amid a significant number of vaccinations and re-vaccinations was observed. It raises the question of the need to study the efficacy of COVID-19 vaccines against this variant. This requires a better understanding of the extent to which the epidemiological efficacy decreases concerning primary infection and hospitalization for vaccinated people during the spread of the Omicron variant.

The Sputnik V vaccine action (Gam-COVID-Vac) implies the use of adenoviral vectors that are not capable of replication in the human body. Yet, these vectors can deliver the SARS-CoV-2 S glycoprotein gene into human cells. Heterologous prime-boost immunization used for the Sputnik V vaccine requires two adenoviruses of different serotypes: Ad26 and Ad5. This makes it possible to recommend a three-week interval between the first and second vaccine doses [15]. The efficacy of the Sputnik V vaccine based on the results of a randomized placebo-controlled trial amounted to 91.6% (95% CI 85.6–95.2%) [1]. The widespread use of the vaccine in Russia, Hungary, Argentina, and Bahrain made it possible to estimate the epidemiological effectiveness of the vaccine in relation to the initial variants of the virus, as well as the Delta variant. Thus, the effectiveness of the first dose of Sputnik V in Argentina was 78.6% (95% CI 74.8–81.7%), three weeks after vaccination. It was estimated during the first five weeks, during the spread of the initial variants of the virus [16]. As for laboratory-confirmed infection and hospitalization in the 60–79-year-old group, the efficacy was 87.6% (95% CI 80.3–92.2%). The effectiveness of first-dose vaccination was estimated in the age group over 60 years during Delta variant dominance. The effectiveness reached 51.98% (95% CI 35.61–64.19%) during the first three months following immunization. At the same time, in the group up to 60 years old, effectiveness was 75.28% (95% CI 69.24–80.13%). Thus, two shots among patients over 60 years old during the period of Delta variant domination is recommended [17].

The national observational study in Hungary HUN-VE (Hungarian Vaccine Efficacy) showed the effectiveness of double immunization with Sputnik V at the level of 85.7% during the period of Alpha variant dominance. It presented 85.7% (95% CI 84.3–86.9%) and 97.5% (95% CI 95.6–98.6%) in protection against infection and death, respectively. These numbers are based on observations during a few months following immunization [18]. The epidemiological efficacy of two doses of Sputnik V was shown in Bahrain for laboratory-confirmed infection and hospitalization [19]. Beta B.1.351, Delta B.1.617.2, and Kappa B.1.617.1 were common during the observation period. According to the data obtained in Russia, in relation to a retrospective cohort study conducted during Delta variant, the effectiveness of Sputnik V was rated at 80% [20].

In a cohort of people living with HIV (PLWH), Sputnik V effectiveness varied. It depended not only on the dominant SARS-CoV-2 variant, but also on the level of CD4+ cells [21]. The effectiveness of Sputnik V among PLWH with CD4 ≥ 350 cells/mL was 81.17% (95% CI 49.13–93.03%) against the original strain. Concerning the Delta variant, it was 65.35% (95% CI 52.61–74.66%). However, the epidemiological efficiency of protection against a severe course of the disease in PLWH with CD4 levels ≥ 350 cells/mL remained at the level of 93.05%.

The effectiveness of Sputnik V against the Omicron variant requires an additional study. The aim of our work is to evaluate the dependence of Sputnik V effectiveness from the number of doses received. We also evaluate the degree of protection in relation to conditions of varying severity.

## 2. Materials and Methods

### 2.1. Research Design and Ethical Issues

The purpose of the present study was to evaluate the effectiveness of the vaccines Sputnik Light, Sputnik V, and additional booster doses during the period of Omicron variant dominance. For this purpose, the random sampling of 1112 patients was conducted at the large City Clinical Hospital No. 67 named after L.A. Vorokhobov of the Moscow City Department (Moscow, Russia). It took place during the period of increasing Omicron incidence, from 11 January to 21 February 2022. Moreover, subsequent monitoring was performed. The only inclusion criterion was a positive PCR test for COVID-19. There was no exclusion criterion. At the time of admission, all hospitalized patients included in the study had nasopharyngeal secretion samples analyzed for the presence of SARS-CoV-2 virus. Their blood serum was also analyzed for the presence of IgG antibodies in NC and RBD antigens. The detection of SARS-CoV-2 RNA was performed by real-time RT-PCR and whole-genome sequencing to differentiate the virus’s genetic line. The severity of the disease was determined by WHO recommendations.

Written informed consent was obtained from all subjects in accordance with the order of the Ministry of Health of the Russian Federation of 21 July 2015 N 474n. All samples were de-identified prior to receipt by the research team. The study was submitted to the Local Ethics Committee of the Gamaleya Center. The committee concluded that the study did not use identifiable biological samples and did not provide any confidential information (decision #14 29 September 2021.).

### 2.2. Sample Collection and RT-PCR

Nasopharyngeal swabs were collected in transport medium for viruses (HEM, catalog number G00155, Russia). Total RNA was extracted from patients’ swabs using a kit for isolation RNA from animal cells/bacteria, smear/scraping of epithelial cells, and viruses with spin-column catalog number RU-250 (Biolabmix, Novosibirsk, Russia). Extracted RNA was tested for SARS-CoV-2 by using a one-step «SARS-CoV-2 FRT» commercial kit with catalog number EA-128 (from N.F. Gamaleya NRCEM, Moscow, Russia). The concentrations of viral samples were determined using in-house plasmid controls with known copy numbers. Specimens with Ct values less than 30 were selected for whole-genome sequencing. In addition, samples were tested for the identification of SARS-CoV-2 Omicron and Delta genetic lines with «AmpliTest SARS-CoV-2 VOC v.3» catalog number K-V017-FRT (from the Centre for Strategic Planning of FMBA, Moscow, Russia).

### 2.3. SARS-CoV-2 Sequencing

#### 2.3.1. Library Preparation and Sequencing

Whole-genome amplification of SARS-CoV-2 virus genome was performed using ARTIC primer V4 with reverse transcription polymerase chain reaction using BioMaster RT-PCR—Premium (Biolabmix, catalog number RM05-200, Russia). DNA libraries were constructed using NEBNext Fast DNA Fragmentation and Library Prep Set for Ion Torrent (New England Biolabs, Ipswich, MA, USA), according to the manufacturer’s instructions. DNA sequencing was performed using Ion 540 Chip and Ion S5XL System (Thermo Fisher Scientific, Waltham, MA, USA).

#### 2.3.2. NGS Data Analysis

The analysis of NGS data was performed, as previously described [22]. Briefly, the quality control of raw data was performed using FastQC [23]. The ARTIC primers were trimmed using cutadapt v3.1 [24]. The trimming of reads by a quality filter was performed using vsearch v2.17.0, and reads smaller than 50 nt were discarded [25]. Trimmed reads were aligned to SARS-CoV-2 Wuhan-Hu-1 (Accession ID GenBank MN908947.3) using BWA-MEM v0.7.17-r1188 [26]. Variant calling and generating consensus sequences were performed using FreeBayes v1.3.5 [27], bcftools v1.12 [28], and bedtools v2.30.0 [29]. Regions with less than 10-fold coverage were masked. Lineages were assigned with Pangolin v3.1.17 using pangoLearn module 2021-12-06. All sequencing data are available online (EPI_ISL_11864996-EPI_ISL_11865125, EPI_ISL_11872910).

### 2.4. Immunological Analysis

IgG antibodies against the nucleocapsid protein (NC) and receptor-binding domain (RBD) were measured using an enzyme immunoassay. A commercial test system «SARS-CoV-2-RBD-ELISA-Gamaleya» (from N.F. Gamaleya NRCEM, Moscow, Russia) was used by the manufacturer’s instructions to detect IgG in the receptor-binding domain of surface glycoprotein S (spike).

To interpret the results, the cut-off value of the optical density OD (cut-off) was calculated using the following formula:

cut-off = OD_K−_ + 3 × SD_K−_

where:

– SD_K−_—mean squared deviation of the optical density of the negative control.

The test serum sample was evaluated as positive if its OD value (A450 mod.) was 2.5 times higher than the cut-off value.

IgG detection of the SARS-CoV-2 (NC) nucleocapsid was performed using an “in-house” ELISA test system. NC was cloned, expressed in *E. coli*, and purified in house. For coating into 96-well ELISA plates (Costar 2592, Corning, NY, USA), 100 μL of a solution of 1 μg/mL of recombinant protein was added to a phosphate-salt buffer (PBS pH 7.4) and incubated overnight at +4 °C. The next day, the plates were blocked for 2 h at room temperature with a S002X buffer containing 0.5% casein (Xema, Moscow, Russia).

The samples were pre-diluted 10 times with an ELISA buffer S011 (Xema, Moscow, Russia). Convalescent sera samples of patients with COVID-19 confirmed by RT-PCR were used as a positive control, and archived sera samples collected before 2020 were used as a negative control.

Before using the ELISA plate, the blocking buffer S002X was removed. The diluted samples were added into the wells of the ELISA plate and incubated for 1 h at 37 °C. Following incubation, the plate was washed 3 times with PBS containing 0.1% Twin-20 and incubated with 100 μL of HRP-conjugated anti-human IgG (Novex A18823; USA) for 1 h at 37 °C. After incubation, the plate was washed 6 times and the reaction was visualized using a chromogen-substrate solution R055 (Xema, Russia). After 10 min, the reaction was stopped with a 10% HCl and the OD of the substrate mi xture was measured on a spectrophotometer at a wavelength of 450 nm (Multiscan FC, Thermo Scientific, MA, USA).

To interpret the results, the cut-off value of the OD was calculated using the following equation:

Cut off = X + 0.2

where:

X is the mean OD value of negative control samples 1 and 2.

For each sample, the positivity coefficient (PC) was calculated using the following formula:

PC = OD sample/cut-off

PC < 0.9—negative, PC > 1.1—positive.

As an additional immunological analysis, the Mindray CL-900i^®^ anti-SARS-CoV-2 IgG (Shenzhen Mindray Bio-Medical Electronics Co., Shenzhen, China) test was used according to the manufacturer’s instructions.

### 2.5. Statistical Analysis

The algorithm for initial data processing and cleaning is shown in Figure S1.

Since the quantitative parameters in most groups varied from the normal distribution (the Shapiro–Wilk and Kolmogorov–Smirnov tests *p* < 0.05), nonparametric parameters were used to describe the median measures of distributions (Me (Q1–Q3)). To compare the parameters in the groups, The Mann–Whitney test or the Kruskal–Wallis test with subsequent posterior comparisons with Bonferroni correction were used.

To compare the qualitative parameters, Pearson’s chi-squared test and Fisher’s exact test were used (explanations are given in the text and captions of the figures). When comparing monopole tables, posteriori comparisons with Benjamin–Hochberg multiplicity correction were used.

The epidemiological effectiveness of vaccination was evaluated by the formula VE = (1 − RR) × 100%, where RR is the risk ratio of becoming sick with various severity in the study groups. Since it was not possible to obtain data on people who were not ill, we used the ratios of vaccinated and ill people in Moscow from open data sources on 26 January 2022 [30,31]. To simplify access to the statistical data from these sources, we converted the Russian language references data to English, and submitted the translation as Supplementary Materials Tables S10 and S11. The 95% confidence interval was calculated according to the previously described method [32].

For statistical analysis, we used R with RStudio (Boston, MA, USA) and SPSS Statistics v.26 (IBM, Armonk, NY, USA).

## 3. Results

We performed the random sampling of patients at the large clinical center in Moscow to assess the effectiveness of the Sputnik Light and Sputnik V vaccines in protection from hospitalization during the prevalence of Omicron. This also included people who received booster doses. Patients were recruited from 11 January to 21 February during the period of increasing COVID-19 incidence in the context of the emerging Omicron variant in Moscow. The only inclusion criterion was a positive COVID-19 PCR test upon admission. For all patients included in the study, nasopharyngeal swabs and blood serum samples were obtained at the time of admission. The severity of COVID-19 was determined on the WHO scale at the time of the analysis and subsequent severity monitoring. PCR analysis to confirm COVID-19 infection was performed. Subsequently, PCR analysis to detect Omicron was also conducted. Then, some samples were sequenced using NGS. These samples confirmed the diagnosis and determined the SARS-CoV-2 variant. Levels of IgG antibodies of NC and RBD were determined for sera samples using ELISA. An impersonal database was formed based on the results of patient recruitment. It included demographic information, vaccination status, laboratory parameters, and the severity of the disease.

The created database with the demographic data of patients was used to analyze the clinical and laboratory data of the patients. The initial sample was 1112 patients. Yet, due to the lack of some data, subsamples were formed for each analysis (Figure S1; Table S1). In total, 240 patients with mild severity, 420—moderate severity, 60—severe, and 78 in critical condition in the intensive care unit (ICU) at the time of performing the analysis were included in the study. The severity groups were homogeneous by gender (*p* = 0.849, Pearson’s chi-squared test), with a slight predominance of women (95% CI 53.8–58.8%) (Table 1).

Mild, severe, and critical groups had no statistically significant differences in age. The median age was 72–74 years. However, the group of moderate course patients included patients that were slightly younger. The median age was 69 years. At the same time, there were more vaccinated patients in this group in comparison to others (*n* = 119, 29.7%). The minimum proportion of vaccinated patients was in the group with patients in a critical condition at the time of sampling (*n* = 11, 14.7%). The differences in the proportions of vaccinated patients among the moderate and critical groups turned out to be statistically significant (*p* = 0.044, chi-squared test following the Benjamin–Hochberg multiplicity correction). Patients who had received other preventive drugs were excluded from the proportion calculations of vaccinated patients.

The age of vaccinated and unvaccinated participants in the study did not vary statistically (*p* = 0.058, Mann–Whitney test). There were more women in the group of unvaccinated patients (*p* = 0.008, Pearson’s chi-squared test) (Table S2). At the same time, a more detailed analysis of the vaccinated group shows that the age of those with two vaccine doses was significantly lower than in the unvaccinated groups or those who received 3–4 doses (Table S3). At the same time, the number of men in the groups increased, depending on the number of doses received. It reached its maximum in the group with 3–4 doses.

From the sample, we obtained information about re-infection cases that was available for 547 people. Only 9 people from these 547 (1.6%) cases were re-infected. Of these, five were in the group of unvaccinated individuals. One patient had previously received Sputnik Light. Three had received Sputnik V. Considering such a low proportion of patients who previously recovered from COVID-19, a special account of the earlier disease was not conducted in further calculations.

### 3.1. Dominant Variants of the Virus and Their Features

For the patients’ enrollment, a PCR analysis was performed using a test system that detected all SARS-CoV-2 variants. To determine the dominant virus variant, in addition to the standard PCR analysis, a test system differentiating between Delta and Omicron was used. Some of the samples with a sufficient viral load (CT below 30) were sequenced. Then, a whole-genome analysis was performed. A differentiating PCR system was used for 620 samples. The severity of the diseases was known in 506 cases from 620, at the time of the analysis. In 506 samples, with the result of a specific PCR analysis, the Delta variant was found in 41 patients, which was 8.1%. At the same time, in the group of patients in a critical condition, the proportion of patients infected with the Delta virus variant was 22.2% (*n* = 10). Table 2 shows a tendency of the increasing proportion of Delta variant infection cases that corresponds to an increase in the severity of the disease. Statistically significant differences were achieved when comparing mild and moderate cases with critical ones.

**Figure 1:**
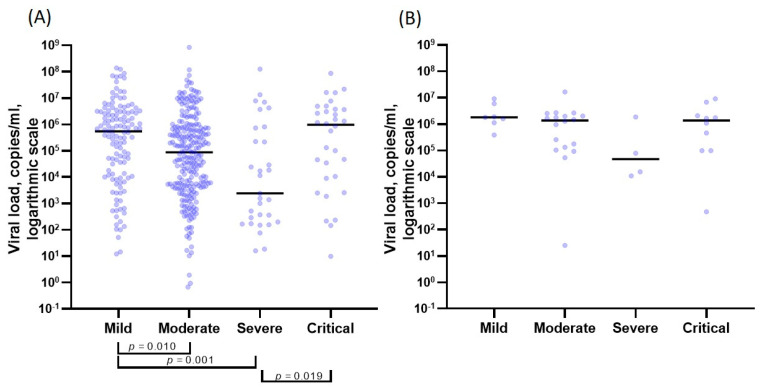
Viral load (copies/mL) depending on the severity of the disease and the strain. (**A**) Omicron strain; (**B**) Delta strain.

The viral load for the Omicron variant tended to decrease in the range from mild to severe forms of the disease. It increased slightly for patients in a critical condition, regardless of the vaccination status. The median viral load values were 5.51 × 10^5^, 8.71 × 10^4^, 2.42 × 10^3^, and 9.63 × 10^5^ copies/mL in the case of mild, moderate, severe, and critical forms, respectively. Statistically significant differences were found between mild and moderate (*p* = 0.010, with Bonferroni multiplicity correction), light and severe (*p* = 0.001, with Bonferroni multiplicity correction), and between severe and critical (*p* = 0.019, with Bonferroni multiplicity correction) forms. The results are presented in Table 3 and Figure 1.

A higher viral load was noted for the Delta variant. However, statistically significant differences were found only for moderate cases. The viral load of the Delta variant in this case was two orders of magnitude higher than for the Omicron variant (the median viral load for the Delta variant was 1.37 × 10^6^, *p* = 0.010, Mann–Whitney test). The Delta variant is characterized by a similar decrease in viral load. Such a decrease corresponds to an increase in severity from mild to severe and an increase in the quantity of critical cases, although, statistically, these differences were not significant (*p* = 0.184, Kruskal–Wallis test). This may be due to the insufficient sample size (Table 3). A significant decrease in viral load in vaccinated patients, compared to unvaccinated patients, was not observed. This applied to those infected with the Delta variant (*p* = 0.111, Mann–Whitney test) and those infected with the Omicron strain (*p* = 0.528, Mann–Whitney test) (Table 4).

**Figure 2:**
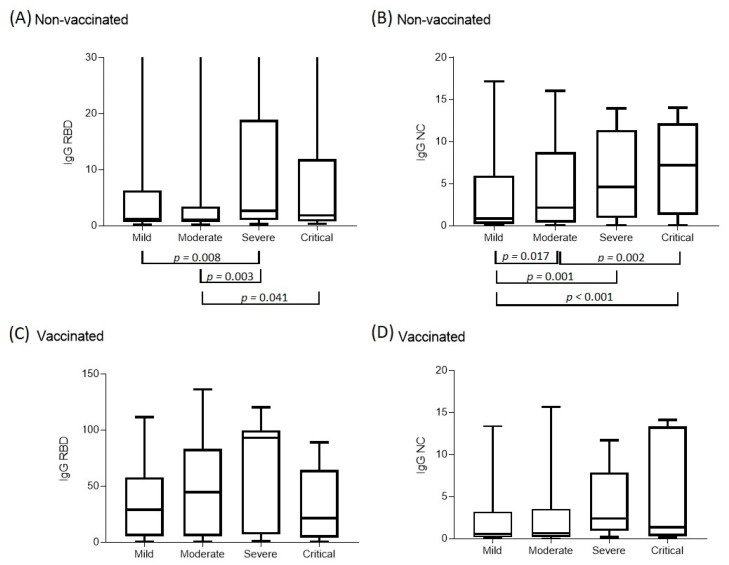
Levels of IgG antibodies in the RBD domain and NC protein depending on severity. Two groups are presented—unvaccinated and vaccinated with two components of Sputnik V. The *Y*-axis indicates the number of antibodies (PC), and shows the median, upper, and lower quantiles. The unvaccinated samples are characterized by a wide variation in the level of IgG antibodies in the RBD domain of the S protein. (**A**)—shows the level of antibodies IgG RBD in unvaccinated patients, (**B**)—the level of IgG NC in unvaccinated patients, (**C**)—the level of IgG RBD in vaccinated patient and (**D**)—the level of IgG NC in vaccinated patients.

**Figure 3:**
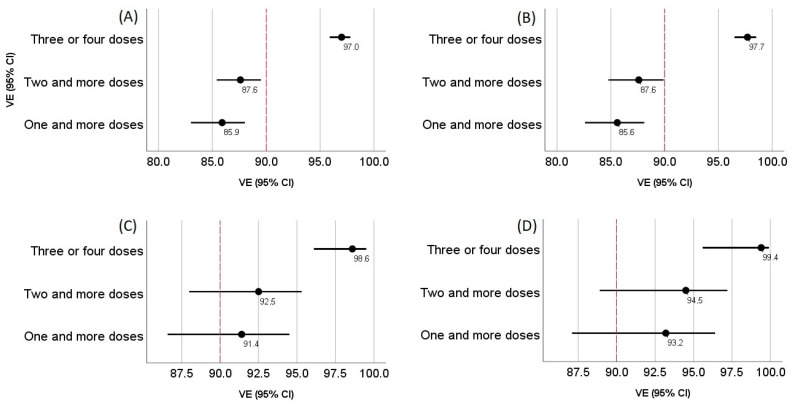
Effectiveness of vaccination in relation to the varying severity of the disease among hospitalized patients, considering the number of vaccine doses received. (**A**) Protection from any cases of hospitalization, (**B**) protection from moderate-severe and more severe cases, (**C**) protection from severe and critical cases, and (**D**) protection from critical cases (VE 95% CI).

We performed whole-genome sequencing of 131 samples to confirm the results of the PCR analysis for samples with a sufficient viral load. According to the obtained data, Omicron was 79.4% in the structure of all variants (*n* = 104) (Figure 2). Line BA.1.1 was the most represented among the subsidiary lines of Omicron, which occurred in 69.2% of cases (*n* = 72). As for PCR, there was a tendency of an increasing proportion of the Delta variant corresponding to the severity of the course (Figure 3, Table 5). Yet, some discrepancy in numbers between the results of the PCR and sequencing are obviously related to the differences in the viral loads of Omicron and Delta. This results in an increase in the latter in genomic sequencing results.

### 3.2. Features of the Immune Response Depending on the Vaccination Status

The ELISA method was used to study the level of IgG antibodies to NC and RBD in different groups, depending on the vaccination status and severity of COVID-19 (Table 4). In unvaccinated patients, there was a tendency of increasing the number of antibodies in both the NC protein and the RBD domain of the SARS-CoV-2 S protein with an increase in the severity of the course of the disease (*p* < 0.001 and *p* = 0.007, respectively). This trend was more pronounced for NC than RBD. In vaccinated patients, there was also a tendency for IgG levels to increase in accordance with the increasing severity of the disease from mild to severe with a further decline in the case of a critical course (Figure 2).

There was a decrease in the number of antibodies, even with a larger number of vaccine components administered to the group of vaccinated patients in a critical condition. This decrease may be due to the time that elapsed after the administration of the final vaccine component (Table S6). Thus, there is a tendency to increase the median time between the last component and the date of hospitalization. The median time escalated from 132 days (4.4 months) for mild cases to 183 (6.0 months) for critical cases. In the case of a critical course of the disease, at least 75% of patients received the final dose of the vaccine more than 120 days (3.9 months) before hospitalization. Statistically, these differences were not significant *p* > 0.05 (*p* = 0.519, Kruskal–Wallis test).

We did not observe any differences in the level of antibodies for patients infected with different variants of the virus (Table S7). The described patterns regarding the increase in the level of antibodies depending on the severity of COVID-19 and the number of vaccine doses administered were confirmed using an alternative immunochemiluminescent analysis of the amount of IgG antibodies to SARS-CoV-2 (Table S8).

### 3.3. Effectiveness of Vaccination with Sputnik Drugs

To assess the effectiveness of the Sputnik vaccine, we used the case–population method that had been recommended by the WHO for situations in which the proportion of vaccinated does not significantly change over time in the population [33]. Data for the number of unvaccinated, vaccinated, and re-vaccinated were used from the open registry.

Epidemiological efficacy was evaluated by the formula VE = (1 − RR) × 100%, where RR is the risk ratio of getting sick with various severity in the study groups. The proportion of vaccinated and non-immune individuals in the population of Moscow was calculated to analyse the epidemiological efficacy of vaccinations (Table 5, Figure 3).

Thus, even though the effectiveness of vaccinations against hospitalization decreased in comparison to the time when other strains were predominant [1,16,17,18,19,20,21], according to our data, it remained at the level of 85.9% (95% CI 83.0–88.0%) for vaccinated patients with at least one component (Table 5). For those who were vaccinated with at least two doses, the epidemiological efficacy rose to 87.6% (95% CI 85.4–89.5%), whereas for those vaccinated with 3 or 4 components (re-vaccination with Sputnik Light or Sputnik V after Sputnik V), it was 97.0% (95% CI 95.9–97.8%).

A lower number of severe course cases among vaccinated people may be due to the higher frequency of the Delta virus variant in this group. We considered that the effectiveness of vaccination against the Delta variant may be greater. Therefore, we conducted a separate analysis of effectiveness. Samples with the Delta virus variant were excluded from the calculation (Table S9). The analysis shows the low impact of the Delta variant residual proportion on the calculation of effectiveness.

## 4. Discussion

According to the international Sputnik vaccine supplier RDIF, the vaccine is registered for use in 71 countries around the world [34]. In the Russian Federation, Argentina, Hungary, Bahrain, and San Marino, the Sputnik vaccine is one of the main vaccines used for COVID-19 prophylaxis. The results of the clinical trials, as well as their effectiveness estimation in field studies, laced Sputnik among the most effective drugs [18,19]. Previously, a very moderate decrease in viral neutralizing activity was shown in relation to the main variants that are of concern. These variants included Alpha, Beta, Gamma, and Delta [35]. Experiments on animal models also did not reveal a significant efficiency drop [36]. This reflects the stability of the protective parameters regarding the main variants of concern.

Meanwhile, the decrease in virus neutralizing activity in matters of the Omicron variant turned out to be significantly more pronounced. Patients, vaccinated with Sputnik V after 6–12 months, had 12 times less virus neutralizing capacity of sera against Omicron compared to original variant B.1.1.1 [16]. At the same time, those who received the vaccine from 0 to 3 months and 3 to 6 months had a virus neutralizing titer detected in 80% and 68.75% of cases, respectively [37]. In turn, the presence of previous infection and booster doses among vaccinated patients increased the proportion of people with a virus neutralizing titer against Omicron to 100% [16,37]. At the same time, it remained unclear to what extent virus neutralization correlates with field effectiveness. Thus, field experiments needed to validate in vitro data obtained using virus-neutralization analysis.

In the case of the current analysis, the virus genetic study showed that the evaluation of the effectiveness mainly relates to the Omicron variant during the period of study (from 11 January to 21 February). More than 90% of infection cases in patients included in the study were caused by the Omicron variant. The composition analysis of the Omicron line showed that 37% was the BA.1 variant, 68%—BA.1.1, and 4%—BA.2, according to the whole-genome sequencing of SARS-CoV-2. The proportion of the Delta variant increased with the severity of the COVID-19 course. While the proportion of the Delta variant among patients with a mild course of the disease was 4.9%, it reached 22.2% (4.5 times more often) among critical-condition patients. This corresponds with the data for the Omicron variant severity. In this data, it is several times milder than the Delta variant [38].

The data on the assessment of IgG antibodies against RBD show that, in all cases with different disease severities, the values in the group of patients vaccinated with two doses were significantly higher than those not vaccinated. Among the vaccinated patients, there was a trend towards an increase in the level of antibodies in RBD corresponding with the increasing severity of the disease, from mild to severe, with a further decline in critical cases (Figure 2). In the case of mild and moderate patients, an increase in the level of antibodies in RBD was observed, depending on the number of components that were administered. This indicates a correlation between the number of vaccine shots and the level of IgG RBD virus-neutralizing antibodies. In this regard, it is expected that an increase in the epidemiological efficacy of the vaccine against the Omicron variant corresponds to an increase in the number of vaccine doses received. Indeed, the epidemiological efficacies regarding protection against hospitalization of patients with COVID-19 in our study were 85.9% (95% CI 83.0–88.0%), 87.6% (95% CI 85.4–89.5%), and 97.0% (95% CI 95.9–97.8%) for those who received more than one, more than two, and more than three injections, respectively. As for the protection from a critical condition requiring ICU, the epidemiological efficacies were 93.2% (95% CI 87.1–96.4%), 94.5% (95% CI 88.9–97.2%), and 99.4% (95% CI 95.6–99.9%) for those who received more than one, more than two, and more than three doses, respectively. The increase in the effectiveness of COVID-19 vaccines in terms of the prevention of more severe forms is well known and characteristic not only of Sputnik, but also of other vaccines, including Pfizer-BioNTech, Moderna, and ChAdOx1 nCoV-19 (AstraZeneca) [18,19]. In the current study, this trend continued for the Omicron variant. This brings hope that a certain level of intense immunity, which is the result of the widespread use of various vaccines based on the original strain’s Spike protein gene, will maintain its effectiveness against new variants of SARS-CoV-2. The emergence of such variants is expected [39]. An alternative to vaccination programs is the isolation and quarantine strategy [40], which is a considerably big-budget plan [41]. Our study indicates that protection against hospitalization and severe forms of COVID-19 remain consistently high for the Sputnik Light and Sputnik V vaccines.

Our research is not without its limitations. In particular, the study is based on observations made in a single medical center. According to Moscow statistics, 10–15% of patients were hospitalized with COVID-19 in the City Clinical Hospital No. 67. Population data for the number of vaccines were obtained from an open register on the date of analysis. Sputnik Light and Sputnik V are the main vaccines used for the prevention of COVID-19 in Russia. The number of doses for other COVID-19 vaccines is more than an order of magnitude lower. However, the register data used did not allow us to include vaccines and booster vaccinations only for Sputnik. In this study, it was also not possible to account for herd immunity and potential undiagnosed cases of both symptomatic and asymptomatic forms of COVID-19, as well as cases of hospitalization with undiagnosed COVID-19. We did not use special methods to optimize our sample. This could result in a slight distortion of the effectiveness calculations. It was also not possible to determine the direct risks of hospitalization for vaccinated and non-vaccinated patients with COVID-19, only the ratio of risks.

## 5. Conclusions

The study shows that vaccination with Sputnik V and Sputnik Light has a high effectiveness for protection against hospitalization. The reduction in the severity of COVID-19 regarding the Omicron variant was also observed. The greatest effectiveness was evident in protection against a critical course of the disease requiring patients to be admitted to the intensive care unit. An increase in the level of antibodies correlated with an increase in the number of doses, ranging from 1 to 4. As a result, there was a lower chance of hospitalization. This should not preclude the need to create new vaccines aimed at SARS-CoV-2 transmission reduction in the population. This suggests that the effectiveness of original vaccines based on the Wuhan S antigen against future VOCs, in terms of protection against hospitalization with Omicron, can be quite high, at least within the recommended vaccination and re-vaccination regimens.

## Figures and Tables

**Table 1:** Demographic analysis of patients (gender, age, and severity are known; *n* = 798).

	Mild, *n* = 240	Moderate, *n* = 420	Severe, *n* = 60	Critical, *n* = 78	*p* Value
Gender Women, *n*, % Men, *n*, %	136 (56.7%) 104 (43.3%)	247 (58.8%) 173 (41.2%)	35 (58.3%) 25 (41.7%)	42 (53.8%) 36 (46.2%)	0.849 (Pearson’s chi-squared test)
Age Me(Q1–Q3)	74 (64–83)	69 (59–76)	72 (63.5–82)	73 (66–82)	<0.001 * p24 = 0.019 * p21 < 0.001 *
Vaccinated (starting with one component)	Taken into account 236 67 (28.4%)	Taken into account 401 119 (29.7%)	Taken into account 59 13 (22.0%)	Taken into account 75 11 (14.7%)	0.043 * (Pearson’s chi-squared test) *p*24 = 0.044 *
Not considered due to not being vaccinated with Sputnik V	4	19	1	3	

*—The differences are statistically significant (*p* < 0.05).

**Table 2:** Genetics of the virus depending on the severity of COVID-19.

	Mild, *n* = 142	Moderate, *n* = 284	Severe, *n* = 35	Critical, *n* = 45	*p*
Delta variant	7 (4.9%)	20 (7.0%)	4 (11.4%)	10 (22.2%)	0.004 * (Fisher’s exact test) *p*14 = 0.002 * p24 = 0.003
Omicron variant	135 (95.1%)	264 (93.0%)	31 (88.6%)	35 (77.8%)	

*—The differences are statistically significant (*p* < 0.05).

**Table 3:** Viral load depending on severity (*n* = 506).

	Mild, *n* = 142	Moderate, *n* = 284	Severe, *n* = 35	Critical, *n* = 45	*p* Value
Viral load of the Omicron variant					
PCR +	127 (94.1%)	247 (93.6%)	31 (100.0%)	32 (91.4%)	
Viral load, Ct Me(Q1–Q3)	26.39 (24.23–31.42)	29.23 (25.78–33.06)	34.53 (27.57–36.76)	26.66 (24.00–32.67)	<0.001 * (the Kruskal–Wallis test) *p*12 = 0.009 * p13 < 0.001 * p43 = 0.020 *
Viral load, copies/mL Me(Q1–Q3)	5.51 × 10^5^ (1.25 × 10^4^–3.03 × 10^6^)	8.71 × 10^4^ (4.64 × 10^3^–7.43 × 10^5^)	2.42 × 10^3^ (2.43 × 10^2^–4.80 × 10^5^)	9.63 × 10^5^ (9.78 × 10^3^– 3.73 × 10^6^ )	<0.001 * (the Kruskal–Wallis test) *p*12 = 0.010 * p13 = 0.001 * p43 = 0.019 *
Viral load of the Delta variant					
PCR+	7 (100.0%)	19 (95.0%)	4 (100.0%)	10 (100.0%)	
Viral load, Ct Me(Q1–Q3)	25.16 (21.02–25.54)	26.22 (24.70–28.63)	30.22 (27.58–31.60)	26.13 (25.23–27.85)	0.063 (the Kruskal–Wallis test)
Viral load, copies/mL Me(Q1–Q3)	1.79 × 10^6^ (1.37 × 10^6^–3.92 × 10^6^)	1.37 × 10^6^ (1.31 × 10^5^–2.00 × 10^6^)	4.71 × 10^4^ (1.33 × 10^4^–9.64 × 10^5^)	1.38 × 10^6^ (9.93 × 10^4^–2.09 × 10^6^)	0.184 (the Kruskal–Wallis test)
*p* (differences between various strains)	0.094 (The Mann–Whitney test)	0.010 * (The Mann–Whitney test)	0.352 (The Mann–Whitney test)	0.805 (The Mann–Whitney test)	

*—The differences are statistically significant (*p* < 0.05).

**Table 4:** Features of the immune response in immunized groups depending on severity. Only unvaccinated and vaccinated patients with at least one component were studied (*n* = 749). Immunological analysis of the coefficient of positivity (PC).

**IgG NC**	**Mild**	**Moderate**	**Severe**	**Critical**	***p***
Unvaccinated Me(Q1–Q3)	*n* = 168 0.86 (0.24–5.97)	*n* = 277 2.16 (0.38–8.77)	*n* = 46 4.61 (1.05–11.09)	*n* = 65 7.22 (1.33–12.18)	<0.001 * (the Kruskal–Wallis test) *p*12 = 0.017 * p13 = 0.001 * p14 < 0.001 * p24 = 0.002 *
Vaccinated with 1 component Me(Q1–Q3)	*n* = 5 0.63 (0.19–0.75)	*n* = 17 2.23 (0.20–12.57)	*n* = 1 --	*n* = 2 5.03 (3.29–6.76)	0.593 (the Kruskal–Wallis test)
Vaccinated with 2 components Me(Q1–Q3)	*n* = 40 0.59 (0.21–3.05)	*n* = 83 0.68 (0.22–3.51)	*n* = 9 2.42 (1.62–6.94)	*n* = 8 1.36 (0.46–13.11)	0.306 (the Kruskal–Wallis test)
Vaccinated with 3–4 components Me(Q1–Q3)	*n* = 22 0.45 (0.23–4.72)	*n* = 19 0.37 (0.16–1.79)	*n* = 3 0.13 (0.11–0.98)	*n* = 1	0.357 (the Kruskal–Wallis test)
*p*	0.682	0.008 * p02 = 0.044 *	0.154	0.788	
**IgG RBD**	**Mild**	**Moderate**	**Severe**	**Critical**	***p***
Unvaccinated Me(Q1–Q3)	*n* = 168 1.17 (0.64–5.9)	*n* = 277 1.08 (0.69–3.22)	*n* = 46 2.66 (1.03–17.79)	*n* = 65 1.89 (0.80–11.93)	0.007 * (the Kruskal–Wallis test) *p*13 =0.008 * p23 = 0.003 * p24 = 0.041 *
Vaccinated with 1 component Me(Q1–Q3)	*n* = 5 32.19 (3.18–72.02)	*n* = 17 3.10 (1.68–24.90)	*n* = 1 --	*n* = 2 69.49 (56.29–82.69)	0.090 (the Kruskal–Wallis test)
Vaccinated with 2 components Me(Q1–Q3)	*n* = 40 29.07 (6.19–57.00)	*n* = 83 44.86 (5.78–83.11)	*n* = 9 93.00 (7.06–95.83)	*n* = 8 21.41 (4.44–57.70)	0.265 (the Kruskal–Wallis test)
Vaccinated with 3–4 components Me(Q1–Q3)	*n* = 22 38.54 (14.56–59.07)	*n* = 19 53.01 (36.39–79.26)	*n* = 3 44.52 (28.66–45.49)	*n* = 1	0.357 (the Kruskal–Wallis test)
*p*	<0.001 * p01 = 0.046 * p02 < 0.001 * p03 < 0.001 *	<0.001 * p02 < 0.001 * p03 < 0.001 *	0.003 * p02 = 0.012 *	0.018 * posteriori comparisons revealed no differences	

*—The differences are statistically significant (*p* < 0.05).

**Table 5:** Analysis of the epidemiological efficacy of vaccination in protection against the severe course of COVID-19 in hospitalized patients.

Quantity of 18+ People in Moscow 10,600,000	Quantity of Vaccinated 6,000,000	Quantity of Previously ill 2,232,836	Quantity of Non-Immune 2,367,164	
	Patients with at least 1 component of vaccine, *n* VE % (95% CI)	Patients with at least 2 components of vaccine, *n* VE % (95% CI)	Patients with at least 3–4 components of vaccine, *n* VE % (95% CI)	Infected and not vaccinated, *n*
Patients with mild and more severe forms of the disease	210 85.9% (95% CI 83.0–88.0%)	185 87.6% (95% CI 85.4–89.5%)	45 97.0% (95% CI 95.9–97.8%)	561
Patients with moderate and more severe forms of the disease	143 85.6% (95% CI 82.6–88.1%)	123 87.6% (95% CI 84.8–89.9%)	23 97.7% (95% CI 96.5–98.5%)	392
Patients with severe and critical forms of the disease	24 91.4% (95% CI 86.6–94.5%)	21 92.5% (95% CI 88.0–95.3%)	4 98.6% (95% CI 96.1–99.5%)	110
Patients in critical condition	11 93.2% (95% CI 87.1–96.4%)	9 94.5% (95% CI 88.9–97.2%)	1 99.4% (95% CI 95.6–99.9%)	64

## Data Availability

All sequence data are available online (EPI_ISL_11864996-EPI_ISL_11865125, EPI_ISL_11872910). Inquiries about access to the original clinical data should be directed to the corresponding authors.

## Supplementary Materials

The following supporting information can be downloaded at: https://www.mdpi.com/article/10.3390/vaccines10060938/s1. Figure S1: Algorithm of data pre-processing; Figure S2: The general distribution of proportions of genetic lines according to the results of SARS-CoV-2 genome sequencing; Figure S3: Distribution of proportions of genetic lines by severity, according to the results of SARS-CoV-2 genome sequencing; Table S1: Patients, included in the study; Table S2: Demographic characteristics according to the vaccination status; Table S3: Demographic characteristics of hospitalized patients according to the number of vaccine doses received; Table S4: Viral load of all severity groups (including patients with unidentified course of the disease) depending on the strain and vaccination status; Table S5: Distribution of SARS-CoV-2 genetic variants by patients’ severity; Table S6: The time elapsed since the injection of the last Gam-Covid-Vac component before hospitalization with COVID-19; Table S7: Viral load and immune response of various virus variants depending on the vaccination status (those vaccinated with drugs other than Gam-Covid-Vac were not included, excluded-4); Table S8: Features of the immune response in immunized groups depending on the severity. Only those who were not vaccinated and those vaccinated with at least one “Gam-Covid-Vac” component were studied (*n* = 749). Immunological analysis for IgG antibodies (Mindray); Table S9: Analysis of the epidemiological efficacy of vaccination in protection against the severe course of COVID-19 in hospitalized patients; Table S10: The number of people vaccinated against COVID-19 in Moscow; Table S11: Coronavirus statistics in Moscow.
