# Supplementary material for: Sputnik V Effectiveness against Hospitalization with COVID-19 during Omicron Dominance
Source: Vaccines (Basel). 2022 Jun 13;10(6):938. doi: 10.3390/vaccines10060938 (PMC9227631; doi:10.3390/vaccines10060938)
Supplement: Supplementary file 1 [file vaccines-10-00938-s001.zip › vaccines-1719160-supplementary.pdf]

vaccines

MDPI

Supplementary materials

Sputnik V effectiveness against hospitalization with COVID-19 during Omicron dominance

Andrey S. Shkoda[1], Vladimir A. Gushchin [2,3,*], Darya A. Ogarkova[2], Svetlana V. Stavitskaya[1], Olga E. Orlova[1], Nadezhda A. Kuznetsova[2], Elena N. Keruntu[1], Andrei A. Pochtovyi [2,3], Alexander V. Pukhov[1], Denis A. Kleymenov[2], Vasyli G. Krzhanovsky[1], Daria V. Vasina[2], Nataliya V. Shkuratova[1], Elena V. Shidlovskaya[2], Alexey L. Gorbunov[1], Daria D. Kustova[2,3], Evgeniya A. Mazurina[1], Sofya R. Kozlova[2], Alexandra V. Soboleva[1], Igor V. Grigoriev[2], Lyudmila L. Pankratyeva[1], Alina S. Odintsova[2,3], Elizaveta D. Belyaeva[2,3], Arina A. Bessonova[2], Lyudmila A. Vasilchenko[2], Igor P. Lupu[2], Ruslan R. Adgamov[2], Artem P. Tkachuk[2], Elizaveta A. Tokarskaya[2], Denis Y. Logunov [2,*] and Alexander L. Gintsburg [2,4]

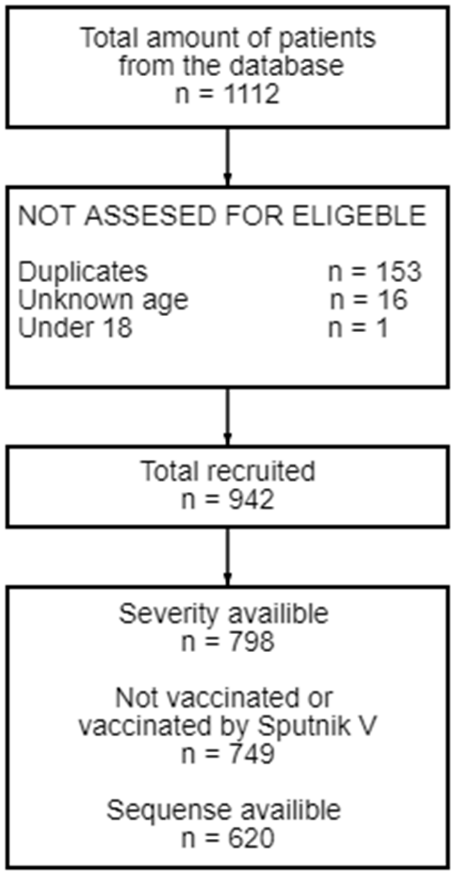

Figure S1. Algorithm of data pre-processing

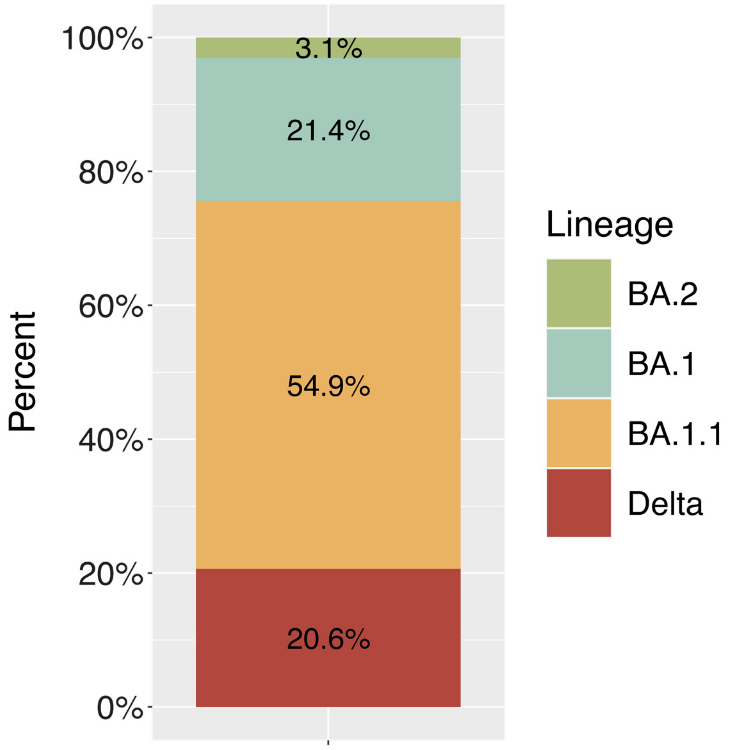

Figure S2. The general distribution of proportions of genetic lines according to the results of SARS-CoV-2 genome sequencing.

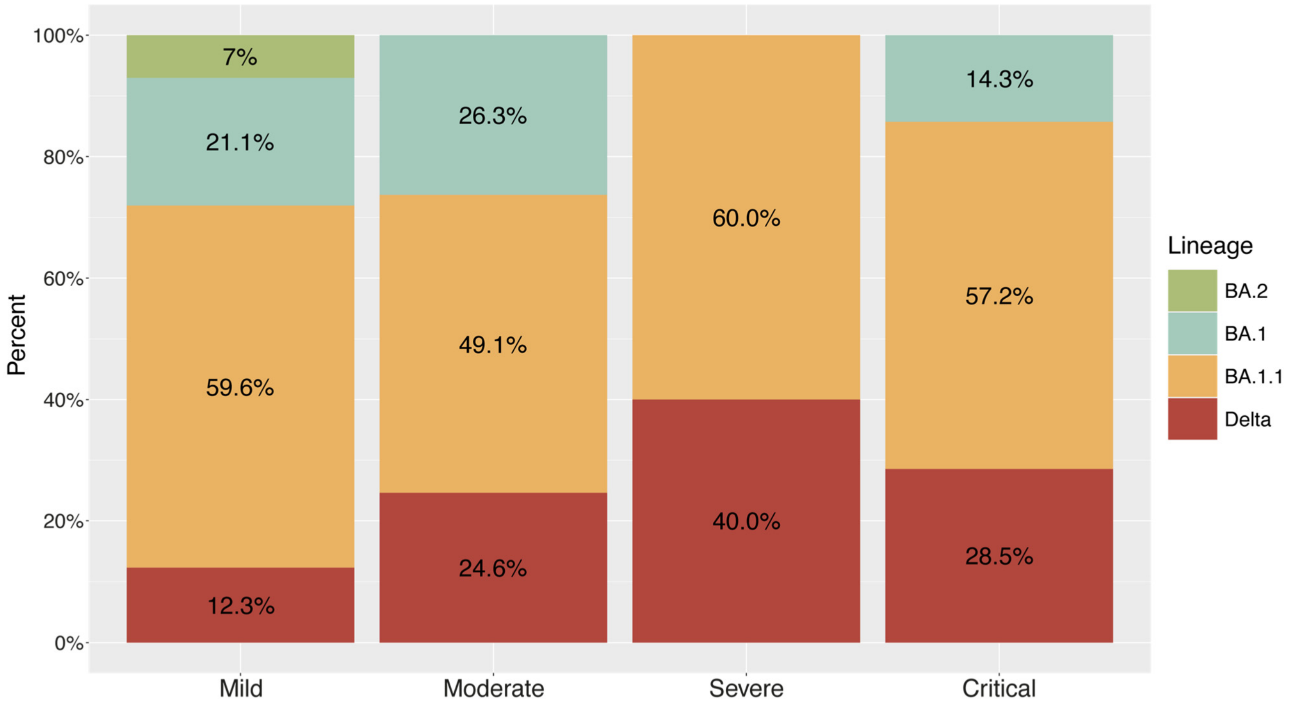

Figure S3. Distribution of proportions of genetic lines by severity, according to the results of SARS-CoV-2 genome sequencing.

Table S1. Patients, included in the study.

Age	942
Gender	942
Known PCR tests<br>of them are positive	942<br>859 (91.2%)
Viral load	858
Immunological analysis (BAU)	889
IgG RBD (known)	934
IgG NC (known)	934
Severity	798
Vaccination	942
Strain	620
	**460**

Table S2. Demographic characteristics according to the vaccination status.

	N	Age, Me[IQR]	Gender:<br>Women, n(%)<br>Men, n (%)
Non-vaccinated	668	72 [62 – 82]	401 (60.0%)<br>267 (40.0%)
Vaccinated	247	70 [61 – 80]	124 (50.2%)<br>123 (49.8%)
P		0.058 (The Mann Whitney test)	0.008* (Pearson's chi-squared test)

Table S3 Demographic characteristics of hospitalized patients according to the number of vaccine doses received.

	N	Age, Me[IQR]	Gender:<br>Women, n(%)<br>Men, n (%)
Non-vaccinated	668	72 [62 – 82]	401 (60.0%)<br>267 (40.0%)
1 component	30	73 [63 – 78]	23 (76.7%)<br>7 (23.3%)
2 components	162	67 [57 – 79]	83 (51.2%)<br>79 (48.8%)
3-4 components	55	74 [69 – 81.5]	18 (32.7%)<br>37 (67.3%)
p		0.001* (the Kraskel-Wallis test)<br>P(non-vaccinated – two doses) = 0.006*<br>P(two doses – three or four doses) = 0.004*	<0.001 (Pearson's chi-squared test)<br>(The proportion of men is higher in the group with more than two vaccine doses received p<0.001, p<0.001, p=0.026)<br><br>In second place-those with two doses received (p = 0.050, p = 0.020)

Table S4. Viral load of all severity groups (including patients with unidentified course of the disease) depending on the strain and vaccination status.

NP	Delta	Omicron	P
Non-vaccinated	N = 36<br>Ct: 25.58[24.69 - 28.39]<br>GE/ml<br>1.63E06[2.61E05 - 2.58E06]	N = 382<br>Ct: 28.33[24.47 - 32.83]<br>GE/ml<br>1.56E05[5.53E03 - 2.26E06]	0.002* (The Mann Whitney test)
Vaccinated with at least 1 component of «Gam-Covid-Vac»	n = 11<br>26.92[25.84 - 28.85]<br>3.86E05 [1.02E05 - 1.63E06]	N = 138<br>Ct: 28.15[25.07 - 32.00]<br>GE/ml<br>1.69E05[6.46E03 - 1.65E06]	0.360 (The Mann Whitney test)
P	0.111 (The Mann Whitney test for the index in copies/ml)	0.528 (The Mann Whitney test for the index in copies/ml)	

Table S5. Distribution of SARS-CoV-2 genetic variants by patients' severity

		Severity			
		Mild	Moderate	Severe	Critical
Delta	n	6	13	1	7
	%	11.5%	24.1%	20.0%	35.0%
Omicron (BA.1)	n	10	15	0	3
	%	19.2%	27.8%	0.0%	15.0%
Omicron (BA.1.1)	n	32	26	4	10
	%	61.5%	48.1%	80.0%	50.0%
Omicron (BA.2)	n	4	0	0	0
	%	7.7%	0.0%	0.0%	0.0%

Table S6. The time elapsed since the injection of the last Gam-Covid-Vac component before hospitalization with Covid-19.

	N (the date of the last vaccination and the date of the analysis are both known)	Me[IQR] time elapsed since vaccination (the last injection) to the date of analysis, days
Mild	66 (98.5%), 1 skip	132 [82 – 184]
Moderate	116 (97.5%), 3 skips	142 [69 – 197.5]
Severe	12 (92.3%), 1 skip	153.5 [96.5 – 240.5]
Critical	11 (100%)	183 [120.5 – 194]

The data was calculated for hospitalized patients with identified severity and at least one vaccine component injected. P = 0.519 (the Kruskal-Wallis test).

Table S7. Viral load and immune response of various virus variants depending on the vaccination status (those vaccinated with drugs other than Gam-Covid-Vac were not included, excluded-4)

	BA.1	BA.1.1	BA.2	Delta	p (the Kruskal - Wallis test)
**Non-vaccinated**	n = 25	n = 58	n = 5	n = 27	
Viral load, Ct Me[IQR]	24.60 [24.22 - 26.46]	**23.49 [22.20 - 25.29]**	25.51 [24.30 - 25.66]	25.32 [23.96 - 26.01]	0.003* $p_{(BA.1.1 - Delta)}$ = 0.022* $p_{(BA.1.1 - BA.1)}$ = 0.014*
Viral load, copies/ml Me[IQR]	2.23E06 [8.03E05 - 4.22E06]	6.13E06 [2.21E06 - 1.61E07]	2.20E06 [1.55E06 - 4.52E06]	1.77E06 [1.37E06 - 4.33E06]	0.012* $p_{(BA.1.1 - BA.1)}$ = 0.023*
IgG NC	0.71 [0.24 - 7.85]	0.78 [0.28 - 5.76]	n =4 0.66 [0.26 - 1.07]	1.25 [0.29 - 11.75]	0.622
IgG RBD	1.05 [0.56 - 16.88]	1.34 [0.73 - 10.74]	n =4 0.51 [0.37 - 1.83]	1.89 [0.95 - 5.55]	0.269
**Vaccinated**	n = 9	n = 23	n = 0	n = 6	
Viral load, Ct Me[IQR]	24.46 [23.04 - 26.64]	n = 22 **24.26 [23.24 - 25.48]**		26.08 [25.16 - 26.92]	0.142
Viral load, copies/ml Me[IQR]	2.54E06 [6.81E05 - 6.79E06]	n = 22 2.91E06 [1.55E06 - 6.39E06]		1.63E06 [4.64E05 - 1.85E06]	0.165
IgG NC	0.61 [0.28 - 2.25]	0.65 [0.24 - 1.76]		0.56 [0.18 - 2.23]	0.956
IgG RBD	8.82 [2.70 - 14.51]	13.54 [2.32 - 43.82]		20.73 [3.10 - 96.54]	0.745

Table S8. Features of the immune response in immunized groups depending on the severity. Only those who were not vaccinated and those vaccinated with at least one "Gam-Covid-Vac" component were studied (n = 749). Immunological analysis for IgG antibodies (Mindray).

	Mild	Moderate	Severe	Critical	P
Non-vaccinated	N = 165<br>2.23 [0.42 – 18.22]	N = 275<br>5.92 [0.91 – 24.49]	N = 44<br>15.93 [1.03 – 93.35]	N = 60<br>24.69 [2.58 – 87.77]	<0.001* (The Kruskal - Wallis test)<br>P14 <0.001*<br>P24 = 0.015*
Vaccinated with 1 «Gam-Covid-Vac» component	N = 5<br>8.05 [0.62 – 17.19]	N = 16<br>6.74 [2.12 – 59.66]	N = 1<br>--	N = 2<br>76.51 [8.39 – 144.63]	0.351 (The Kruskal - Wallis test)
Vaccinated with 2 «Gam-Covid-Vac» components	N = 40<br>8.18 [2.12 – 58.04]	N = 80<br>19.56 [5.59 – 46.56]	N = 9<br>48.54 [12.29 – 124.21]	N = 8<br>5.04 [1.10 – 163.28]	0.389 (The Kruskal - Wallis test)
Vaccinated with 3-4 «Gam-Covid-Vac» components	N = 21<br>28.92 [12.81 – 38.47]	N = 19<br>24.39 [12.92 – 56.92]	N =3<br>5.12 [3.49 – 12.71]	N = 1	0.204 (The Kruskal - Wallis test)
P	<0.001*<br>P02 = 0.012*<br>P03<0.001*	<0.001*<br>P02 < 0.001*<br>P03=0.004*	0.254	0.823	

Table S9. Analysis of the epidemiological efficacy of vaccination in protection against the severe course of COVID-19 in hospitalized patients (Omicron strain only, one known case of reinfection was deleted). 448 patients were studied.

**Quantity of 18+ people in Moscow**<br><br>**10 600 000**	**Quantity of vaccinated**<br><br>**6 000 000**	**Quantity of previously ill**<br><br>**2 232 836**	**Quantity of "non-immune"**<br><br>**2 367 164**	
	Got infected and had no less that 1 component of Gam-Covid-Vac,<br><br>N<br><br>VE % (95% CI)	Got infected and had no less that 2 components of Gam-Covid-Vac,<br><br>N<br><br>VE % (95% CI)	Got infected and had at no less than 3-4 components of Gam-Covid-Vac,<br><br>N<br><br>VE % (95% CI)	Non-vaccinated got infected<br><br>N
Patients with mild and more severe forms of the disease	115<br><br>86.4%<br><br>(95% CI 83.2 - 89.0%)	99<br><br>88.3%<br><br>(95% CI 85.3 - 90.6%)	30<br><br>96.4%<br><br>(95% CI 94.8 - 97.6%)	333
Patients with moderate and more severe forms of the disease	80<br><br>86.6%<br><br>(95% CI 82.7 - 89.6%)	68<br><br>88.6%<br><br>(95% CI 85.1 - 91.3%)	17<br><br>97.1%<br><br>(95% CI 95.3 - 98.3%)	235
Patients with severe and critical forms of the disease	10<br><br>92.8%<br><br>(95% CI 85.9 - 96.3%)	9<br><br>93.5%<br><br>(95% CI 86.9 - 96.8%)	3<br><br>97.8%<br><br>(95% CI 93.1 - 99.3%)	55
Patients in critical condition	6<br><br>91.8%<br><br>(95% CI 80.3 - 96.6%)	5<br><br>93.2%<br><br>(95% CI 82.4 - 97.4%)	1<br><br>98.6%<br><br>(95% CI 90.0 - 99.8%)	29

Table S10. The number of people vaccinated against COVID-19 in Moscow

Reference https://gogov.ru/covid-v-stats/msk#data

Cumulative total

For Date	Vaccinated, person	Fully vaccinated	Revaccinated
11 February 2022	6 199 585	5 803 415	
4 February 2022	6 149 637	5 756 593	
28 January 2022	6 062 844	5 673 588	
26 January 2022	6 000 000		1 000 000
1 January 2022	5 900 000		
17 December 2021	5 815 552	5 467 395	
10 December 2021	5 815 552	5 296 362	
6 December 2021	5 700 000		680 000
3 December 2021	5 637 147	5 072 241	
26 November 2021	5 502 513	4 926 308	
19 November 2021	5 330 506	4 788 247	750 000
4 November 2021	5 051 629	4 410 000	
22 октября 2021	5 051 629		
14 октября 2021	5 000 000		279 000
5 октября 2021	5 000 000		
13 September 2021	5 000 000		118 000
11 August 2021	4 539 902	3 611 289	
8 August 2021	4 479 278	3 556 053	
1 August 2021	4 381 085	3 194 373	
25 Jule 2021	4 270 379	2 694 832	
20 Jule 2021	4 000 000	2 200 000	
16 Jule 2021	3 800 000	2 000 000	
8 Jule 2021	3 200 000		
7 Jule 2021	3 100 000	1 800 000	
5 Jule 2021	3 000 000	1 800 000	
3 Jule 2021	2 700 000		
2 Jule 2021	2 600 000		
26 June 2021	2 000 000	1 600 000	
22 June 2021	2 000 000		
18 June 2021	1 800 000	1 500 000	
16 June 2021	1 800 000		
26 May 2021	1 500 000		
18 May 2021	1 400 000	1 300 000	

14 May 2021	1 300 000	
15 April 2021	1 000 000	820 000
26 March 2021	1 000 000	800 000
25 March 2021	1 000 000	
17 March 2021	700 000	
15 March 2021	700 000	
5 March 2021	700 000	
4 March 2021	600 000	
26 February 2021	600 000	
18 February 2021	600 000	
17 February 2021	555 000	
11 February 2021	500 000	
4 February 2021	400 000	
28 January 2021	320 000	
21 January 2021	220 000	
19 January 2021	200 000	
18 January 2021	190 000	
14 January 2021	140 000	
1 January 2021	50 000	

Table S11. Coronavirus statistics in Moscow

Reference	https://gogov.ru/covid-19/msk#data		
**Date**	**Cases**	**Deaths**	**Recoveries**
07.06.2022	2 772 365 *(+167)*	43 824 *(+11)*	2 581 598
06.06.2022	2 772 198 *(+189)*	43 813 *(+14)*	2 581 254
05.06.2022	2 772 009 *(+185)*	43 799 *(+13)*	2 581 043
04.06.2022	2 771 824 *(+272)*	43 786 *(+15)*	2 580 895
03.06.2022	2 771 552 *(+264)*	43 771 *(+14)*	2 580 581
02.06.2022	2 771 288 *(+353)*	43 757 *(+10)*	2 580 293
01.06.2022	2 770 935 *(+367)*	43 747 *(+13)*	2 580 022
31.05.2022	2 770 568 *(+190)*	43 734 *(+14)*	2 579 638
30.05.2022	2 770 378 *(+191)*	43 720 *(+12)*	2 579 330
29.05.2022	2 770 187 *(+286)*	43 708 *(+11)*	2 579 114
28.05.2022	2 769 901 *(+267)*	43 697 *(+13)*	2 578 937
27.05.2022	2 769 634 *(+319)*	43 684 *(+12)*	2 578 716
26.05.2022	2 769 315 *(+368)*	43 672 *(+14)*	2 578 355
25.05.2022	2 768 947 *(+370)*	43 658 *(+17)*	2 578 023
24.05.2022	2 768 577 *(+172)*	43 641 *(+15)*	2 577 664
23.05.2022	2 768 405 *(+229)*	43 626 *(+14)*	2 577 305
22.05.2022	2 768 176 *(+278)*	43 612 *(+13)*	2 577 050
21.05.2022	2 767 898 *(+337)*	43 599 *(+15)*	2 576 765
20.05.2022	2 767 561 *(+368)*	43 584 *(+14)*	2 576 412
19.05.2022	2 767 193 *(+365)*	43 570 *(+16)*	2 575 983
18.05.2022	2 766 828 *(+380)*	43 554 *(+17)*	2 575 553
17.05.2022	2 766 448 *(+212)*	43 537 *(+19)*	2 575 194
16.05.2022	2 766 236 *(+288)*	43 518 *(+18)*	2 574 946
15.05.2022	2 765 948 *(+335)*	43 500 *(+14)*	2 574 691
14.05.2022	2 765 613 *(+402)*	43 486 *(+16)*	2 574 424
13.05.2022	2 765 211 *(+474)*	43 470 *(+15)*	2 574 079
12.05.2022	2 764 737 *(+294)*	43 455 *(+18)*	2 573 636
11.05.2022	2 764 443 *(+217)*	43 437 *(+16)*	2 573 152
10.05.2022	2 764 226 *(+237)*	43 421 *(+17)*	2 572 757
09.05.2022	2 763 989 *(+268)*	43 404 *(+19)*	2 572 477
08.05.2022	2 763 721 *(+396)*	43 385 *(+16)*	2 572 227
07.05.2022	2 763 325 *(+387)*	43 369 *(+18)*	2 571 904
06.05.2022	2 762 938 *(+526)*	43 351 *(+19)*	2 571 492
05.05.2022	2 762 412 *(+393)*	43 332 *(+16)*	2 570 999
04.05.2022	2 762 019 *(+282)*	43 316 *(+19)*	2 570 377

03.05.2022	2 761 737 (+250)	43 297 (+18)	2 569 856
02.05.2022	2 761 487 (+334)	43 279 (+17)	2 569 374
01.05.2022	2 761 153 *(+455)*	43 262 *(+19)*	2 568 898
30.04.2022	2 760 698 *(+485)*	43 243 *(+18)*	2 568 474
29.04.2022	2 760 213 (+570)	43 225 (+17)	2 567 858
28.04.2022	2 759 643 (+629)	43 208 (+18)	2 567 229
27.04.2022	2 759 014 (+760)	43 190 (+16)	2 566 693
26.04.2022	2 758 254 (+378)	43 174 (+19)	2 566 156
25.04.2022	2 757 876 (+455)	43 155 (+17)	2 565 312
24.04.2022	2 757 421 *(+584)*	43 138 *(+18)*	2 564 838
23.04.2022	2 756 837 *(+602)*	43 120 *(+15)*	2 564 391
22.04.2022	2 756 235 (+644)	43 105 (+16)	2 563 696
21.04.2022	2 755 591 (+616)	43 089 (+18)	2 562 943
20.04.2022	2 754 975 (+749)	43 071 (+19)	2 562 329
19.04.2022	2 754 226 (+416)	43 052 (+18)	2 561 601
18.04.2022	2 753 810 (+497)	43 034 (+17)	2 560 708
17.04.2022	2 753 313 *(+603)*	43 017 *(+19)*	2 560 043
16.04.2022	2 752 710 *(+634)*	42 998 *(+16)*	2 559 246
15.04.2022	2 752 076 (+766)	42 982 (+18)	2 558 422
14.04.2022	2 751 310 (+868)	42 964 (+17)	2 557 511
13.04.2022	2 750 442 (+933)	42 947 (+19)	2 556 555
12.04.2022	2 749 509 (+489)	42 928 (+18)	2 555 538
11.04.2022	2 749 020 (+589)	42 910 (+19)	2 554 544
10.04.2022	2 748 431 *(+742)*	42 891 *(+18)*	2 553 846
09.04.2022	2 747 689 *(+801)*	42 873 *(+17)*	2 553 278
08.04.2022	2 746 888 (+855)	42 856 (+19)	2 552 447
07.04.2022	2 746 033 (+1 004)	42 837 (+20)	2 551 635
06.04.2022	2 745 029 (+1 072)	42 817 (+18)	2 550 593
05.04.2022	2 743 957 (+596)	42 799 (+21)	2 549 372
04.04.2022	2 743 361 (+698)	42 778 (+20)	2 548 262
03.04.2022	2 742 663 *(+835)*	42 758 *(+22)*	2 547 527
02.04.2022	2 741 828 *(+994)*	42 736 *(+21)*	2 546 782
01.04.2022	2 740 834 (+1 038)	42 715 (+19)	2 545 875
31.03.2022	2 739 796 (+1 130)	42 696 (+22)	2 544 974
30.03.2022	2 738 666 (+1 165)	42 674 (+21)	2 543 959
29.03.2022	2 737 501 (+708)	42 653 (+23)	2 542 802
28.03.2022	2 736 793 (+703)	42 630 (+20)	2 541 469
27.03.2022	2 736 090 *(+867)*	42 610 *(+22)*	2 540 464
26.03.2022	2 735 223 *(+925)*	42 588 *(+23)*	2 539 938

25.03.2022	2 734 298 *(+1 086)*	42 565 *(+25)*	2 539 045
24.03.2022	2 733 212 *(+1 163)*	42 540 *(+26)*	2 538 159
23.03.2022	2 732 049 *(+1 306)*	42 514 *(+28)*	2 537 114
22.03.2022	2 730 743 *(+723)*	42 486 *(+30)*	2 535 852
21.03.2022	2 730 020 *(+771)*	42 456 *(+29)*	2 534 143
20.03.2022	2 729 249 *(+928)*	42 427 *(+31)*	2 533 045
19.03.2022	2 728 321 *(+927)*	42 396 *(+30)*	2 532 057
18.03.2022	2 727 394 *(+1 130)*	42 366 *(+38)*	2 530 464
17.03.2022	2 726 264 *(+1 352)*	42 328 *(+42)*	2 528 385
16.03.2022	2 724 912 *(+1 621)*	42 286 *(+40)*	2 526 380
15.03.2022	2 723 291 *(+999)*	42 246 *(+41)*	2 524 854
14.03.2022	2 722 292 *(+957)*	42 205 *(+44)*	2 523 496
13.03.2022	2 721 335 *(+1 437)*	42 161 *(+49)*	2 522 435
12.03.2022	2 719 898 *(+1 655)*	42 112 *(+52)*	2 521 223
11.03.2022	2 718 243 *(+2 102)*	42 060 *(+58)*	2 519 398
10.03.2022	2 716 141 *(+1 215)*	42 002 *(+57)*	2 517 165
09.03.2022	2 714 926 *(+1 292)*	41 945 *(+59)*	2 514 574
08.03.2022	2 713 634 *(+1 151)*	41 886 *(+67)*	2 511 907
07.03.2022	2 712 483 *(+1 542)*	41 819 *(+65)*	2 508 951
06.03.2022	2 710 941 *(+1 901)*	41 754 *(+75)*	2 506 536
05.03.2022	2 709 040 *(+2 060)*	41 679 *(+77)*	2 504 198
04.03.2022	2 706 980 *(+2 463)*	41 602 *(+79)*	2 501 129
03.03.2022	2 704 517 *(+2 683)*	41 523 *(+82)*	2 497 491
02.03.2022	2 701 834 *(+3 024)*	41 441 *(+81)*	2 492 797
01.03.2022	2 698 810 *(+2 066)*	41 360 *(+84)*	2 487 594
28.02.2022	2 696 744 *(+2 584)*	41 276 *(+80)*	2 481 921
27.02.2022	2 694 160 *(+3 050)*	41 196 *(+81)*	2 477 563
26.02.2022	2 691 110 *(+3 947)*	41 115 *(+83)*	2 473 013
25.02.2022	2 687 163 *(+3 333)*	41 032 *(+84)*	2 466 755
24.02.2022	2 683 830 *(+4 730)*	40 948 *(+82)*	2 459 296
23.02.2022	2 679 100 *(+5 664)*	40 866 *(+83)*	2 452 895
22.02.2022	2 673 436 *(+4 250)*	40 783 *(+85)*	2 443 899
21.02.2022	2 669 186 *(+4 997)*	40 698 *(+83)*	2 431 079
20.02.2022	2 664 189 *(+6 388)*	40 615 *(+84)*	2 418 457
19.02.2022	2 657 801 *(+6 874)*	40 531 *(+87)*	2 398 976
18.02.2022	2 650 927 *(+8 308)*	40 444 *(+86)*	2 363 212
17.02.2022	2 642 619 *(+9 705)*	40 358 *(+85)*	2 324 231
16.02.2022	2 632 914 *(+11 262)*	40 273 *(+87)*	2 281 792
15.02.2022	2 621 652 *(+8 160)*	40 186 *(+84)*	2 244 013

14.02.2022	2 613 492 *(+9 616)*	40 102 *(+82)*	2 214 046
13.02.2022	2 603 876 *(+12 496)*	40 020 *(+86)*	2 185 131
12.02.2022	2 591 380 *(+14 336)*	39 934 *(+85)*	2 160 558
11.02.2022	2 577 044 *(+18 541)*	39 849 *(+81)*	2 140 512
10.02.2022	2 558 503 *(+22 747)*	39 768 *(+84)*	2 118 897
09.02.2022	2 535 756 *(+11 521)*	39 684 *(+82)*	2 094 533
08.02.2022	2 524 235 *(+12 902)*	39 602 *(+85)*	2 068 597
07.02.2022	2 511 333 *(+15 442)*	39 517 *(+83)*	2 047 924
06.02.2022	2 495 891 *(+18 856)*	39 434 *(+81)*	2 034 827
05.02.2022	2 477 035 *(+22 444)*	39 353 *(+82)*	2 021 805
04.02.2022	2 454 591 *(+25 019)*	39 271 *(+79)*	2 007 965
03.02.2022	2 429 572 *(+26 904)*	39 192 *(+76)*	1 994 160
02.02.2022	2 402 668 *(+23 784)*	39 116 *(+78)*	1 981 801
01.02.2022	2 378 884 *(+21 533)*	39 038 *(+76)*	1 970 326
31.01.2022	2 357 351 *(+23 417)*	38 962 *(+75)*	1 962 382
30.01.2022	2 333 934 *(+24 030)*	38 887 *(+77)*	1 956 577
29.01.2022	2 309 904 *(+26 488)*	38 810 *(+76)*	1 951 552
28.01.2022	2 283 416 *(+23 994)*	38 734 *(+74)*	1 946 142
27.01.2022	2 259 422 *(+26 586)*	38 660 *(+72)*	1 940 819
26.01.2022	2 232 836 *(+19 856)*	38 588 *(+74)*	1 935 354
25.01.2022	2 212 980 *(+18 935)*	38 514 *(+75)*	1 931 016
24.01.2022	2 194 045 *(+19 509)*	38 439 *(+71)*	1 927 017
23.01.2022	2 174 536 *(+17 528)*	38 368 *(+73)*	1 923 786
22.01.2022	2 157 008 *(+16 094)*	38 295 *(+69)*	1 920 971
21.01.2022	2 140 914 *(+15 987)*	38 226 *(+72)*	1 917 946
20.01.2022	2 124 927 *(+11 557)*	38 154 *(+70)*	1 915 633
19.01.2022	2 113 370 *(+8 795)*	38 084 *(+71)*	1 913 326
18.01.2022	2 104 575 *(+8 342)*	38 013 *(+69)*	1 911 329
17.01.2022	2 096 233 *(+7 529)*	37 944 *(+67)*	1 909 491
16.01.2022	2 088 704 *(+6 480)*	37 877 *(+70)*	1 907 686
15.01.2022	2 082 224 *(+6 555)*	37 807 *(+68)*	1 905 315
14.01.2022	2 075 669 *(+5 712)*	37 739 *(+69)*	1 902 438
13.01.2022	2 069 957 *(+5 490)*	37 670 *(+71)*	1 899 550
12.01.2022	2 064 467 *(+4 008)*	37 599 *(+66)*	1 896 693
11.01.2022	2 060 459 *(+4 635)*	37 533 *(+68)*	1 893 867
10.01.2022	2 055 824 *(+2 778)*	37 465 *(+70)*	1 891 175
09.01.2022	2 053 046 *(+3 274)*	37 395 *(+71)*	1 889 078
08.01.2022	2 049 772 *(+3 072)*	37 324 *(+69)*	1 887 020
07.01.2022	2 046 700 *(+3 694)*	37 255 *(+72)*	1 884 960

06.01.2022	2 043 006 *(+2 357)*	37 183 *(+67)*	1 882 737
05.01.2022	2 040 649 *(+2 307)*	37 116 *(+71)*	1 880 499
04.01.2022	2 038 342 *(+1 967)*	37 045 *(+68)*	1 877 851
03.01.2022	2 036 375 *(+1 335)*	36 977 *(+70)*	1 875 577
02.01.2022	2 035 040 *(+1 824)*	36 907 *(+69)*	1 873 286
01.01.2022	2 033 216 *(+2 472)*	36 838 *(+67)*	1 871 054
31.12.2021	2 030 744 *(+2 739)*	36 771 *(+69)*	1 868 696
30.12.2021	2 028 005 *(+2 661)*	36 702 *(+71)*	1 865 970
29.12.2021	2 025 344 *(+1 798)*	36 631 *(+70)*	1 863 475
28.12.2021	2 023 546 *(+1 705)*	36 561 *(+73)*	1 860 822
27.12.2021	2 021 841 *(+2 239)*	36 488 *(+68)*	1 857 711
26.12.2021	2 019 602 *(+2 180)*	36 420 *(+71)*	1 855 083
25.12.2021	2 017 422 *(+2 714)*	36 349 *(+72)*	1 852 320
24.12.2021	2 014 708 *(+2 126)*	36 277 *(+75)*	1 849 212
23.12.2021	2 012 582 *(+3 062)*	36 202 *(+77)*	1 846 440
22.12.2021	2 009 520 *(+1 904)*	36 125 *(+81)*	1 843 148
21.12.2021	2 007 616 *(+1 906)*	36 044 *(+80)*	1 840 029
20.12.2021	2 005 710 *(+2 630)*	35 964 *(+78)*	1 836 874
19.12.2021	2 003 080 *(+3 013)*	35 886 *(+81)*	1 834 232
18.12.2021	2 000 067 *(+2 283)*	35 805 *(+79)*	1 831 571
17.12.2021	1 997 784 *(+2 755)*	35 726 *(+83)*	1 828 635
16.12.2021	1 995 029 *(+3 322)*	35 643 *(+85)*	1 825 126
15.12.2021	1 991 707 *(+2 459)*	35 558 *(+87)*	1 822 086
14.12.2021	1 989 248 *(+1 976)*	35 471 *(+89)*	1 819 020
13.12.2021	1 987 272 *(+2 894)*	35 382 *(+84)*	1 815 584
12.12.2021	1 984 378 *(+2 862)*	35 298 *(+88)*	1 812 568
11.12.2021	1 981 516 *(+3 140)*	35 210 *(+86)*	1 809 074
10.12.2021	1 978 376 *(+3 477)*	35 124 *(+89)*	1 805 490
09.12.2021	1 974 899 *(+2 948)*	35 035 *(+92)*	1 802 118
08.12.2021	1 971 951 *(+3 004)*	34 943 *(+90)*	1 798 947
07.12.2021	1 968 947 *(+2 573)*	34 853 *(+94)*	1 795 940
06.12.2021	1 966 374 *(+3 134)*	34 759 *(+91)*	1 792 971
05.12.2021	1 963 240 *(+3 301)*	34 668 *(+92)*	1 790 218
04.12.2021	1 959 939 *(+3 356)*	34 576 *(+93)*	1 787 034
03.12.2021	1 956 583 *(+3 304)*	34 483 *(+95)*	1 783 316
02.12.2021	1 953 279 *(+3 805)*	34 388 *(+92)*	1 779 716
01.12.2021	1 949 474 *(+2 757)*	34 296 *(+94)*	1 776 082
30.11.2021	1 946 717 *(+2 788)*	34 202 *(+97)*	1 772 354
29.11.2021	1 943 929 *(+3 434)*	34 105 *(+93)*	1 768 538

28.11.2021	1 940 495 *(+3 086)*	34 012 *(+95)*	1 764 835
27.11.2021	1 937 409 *(+3 430)*	33 917 *(+96)*	1 760 697
26.11.2021	1 933 979 *(+3 929)*	33 821 *(+94)*	1 755 581
25.11.2021	1 930 050 *(+3 349)*	33 727 *(+92)*	1 750 445
24.11.2021	1 926 701 *(+2 533)*	33 635 *(+93)*	1 745 284
23.11.2021	1 924 168 *(+2 749)*	33 542 *(+96)*	1 739 891
22.11.2021	1 921 419 *(+2 786)*	33 446 *(+94)*	1 734 540
21.11.2021	1 918 633 *(+3 438)*	33 352 *(+97)*	1 729 001
20.11.2021	1 915 195 *(+3 239)*	33 255 *(+93)*	1 722 920
19.11.2021	1 911 956 *(+3 371)*	33 162 *(+95)*	1 716 433
18.11.2021	1 908 585 *(+4 062)*	33 067 *(+94)*	1 709 871
17.11.2021	1 904 523 *(+2 966)*	32 973 *(+96)*	1 702 667
16.11.2021	1 901 557 *(+2 606)*	32 877 *(+97)*	1 695 190
15.11.2021	1 898 951 *(+3 789)*	32 780 *(+93)*	1 687 803
14.11.2021	1 895 162 *(+3 734)*	32 687 *(+95)*	1 681 439
13.11.2021	1 891 428 *(+4 185)*	32 592 *(+97)*	1 674 966
12.11.2021	1 887 243 *(+5 183)*	32 495 *(+96)*	1 668 369
11.11.2021	1 882 060 *(+6 240)*	32 399 *(+94)*	1 661 417
10.11.2021	1 875 820 *(+3 927)*	32 305 *(+98)*	1 655 912
09.11.2021	1 871 893 *(+5 287)*	32 207 *(+95)*	1 648 363
08.11.2021	1 866 606 *(+4 982)*	32 112 *(+97)*	1 641 106
07.11.2021	1 861 624 *(+4 975)*	32 015 *(+96)*	1 635 844
06.11.2021	1 856 649 *(+6 880)*	31 919 *(+98)*	1 630 606
05.11.2021	1 849 769 *(+6 407)*	31 821 *(+93)*	1 624 648
04.11.2021	1 843 362 *(+6 305)*	31 728 *(+97)*	1 618 624
03.11.2021	1 837 057 *(+6 827)*	31 631 *(+95)*	1 611 991
02.11.2021	1 830 230 *(+5 736)*	31 536 *(+98)*	1 605 363
01.11.2021	1 824 494 *(+7 103)*	31 438 *(+96)*	1 599 004
31.10.2021	1 817 391 *(+7 603)*	31 342 *(+94)*	1 593 747
30.10.2021	1 809 788 *(+7 267)*	31 248 *(+97)*	1 588 697
29.10.2021	1 802 521 *(+7 511)*	31 151 *(+93)*	1 583 191
28.10.2021	1 795 010 *(+8 440)*	31 058 *(+95)*	1 578 057
27.10.2021	1 786 570 *(+5 789)*	30 963 *(+91)*	1 572 825
26.10.2021	1 780 781 *(+6 074)*	30 872 *(+86)*	1 568 354
25.10.2021	1 774 707 *(+7 778)*	30 786 *(+83)*	1 564 430
24.10.2021	1 766 929 *(+5 279)*	30 703 *(+84)*	1 560 538
23.10.2021	1 761 650 *(+7 803)*	30 619 *(+82)*	1 556 721
22.10.2021	1 753 847 *(+8 166)*	30 537 *(+79)*	1 552 735
21.10.2021	1 745 681 *(+7 897)*	30 458 *(+77)*	1 548 573

20.10.2021	1 737 784 *(+5 847)*	30 381 *(+76)*	1 544 301
19.10.2021	1 731 937 *(+5 700)*	30 305 *(+73)*	1 540 144
18.10.2021	1 726 237 *(+6 823)*	30 232 *(+69)*	1 536 156
17.10.2021	1 719 414 *(+6 740)*	30 163 *(+70)*	1 532 919
16.10.2021	1 712 674 *(+6 545)*	30 093 *(+72)*	1 529 713
15.10.2021	1 706 129 *(+6 631)*	30 021 *(+70)*	1 526 076
14.10.2021	1 699 498 *(+6 712)*	29 951 *(+71)*	1 522 320
13.10.2021	1 692 786 *(+4 410)*	29 880 *(+72)*	1 519 184
12.10.2021	1 688 376 *(+4 699)*	29 808 *(+70)*	1 516 617
11.10.2021	1 683 677 *(+5 002)*	29 738 *(+71)*	1 514 140
10.10.2021	1 678 675 *(+4 610)*	29 667 *(+72)*	1 511 856
09.10.2021	1 674 065 *(+6 001)*	29 595 *(+70)*	1 509 679
08.10.2021	1 668 064 *(+4 595)*	29 525 *(+71)*	1 507 468
07.10.2021	1 663 469 *(+5 404)*	29 454 *(+69)*	1 505 180
06.10.2021	1 658 065 *(+3 589)*	29 385 *(+71)*	1 502 833
05.10.2021	1 654 476 *(+3 827)*	29 314 *(+72)*	1 500 434
04.10.2021	1 650 649 *(+4 410)*	29 242 *(+66)*	1 498 183
03.10.2021	1 646 239 *(+4 294)*	29 176 *(+65)*	1 496 227
02.10.2021	1 641 945 *(+4 032)*	29 111 *(+67)*	1 494 342
01.10.2021	1 637 913 *(+3 993)*	29 044 *(+64)*	1 492 226
30.09.2021	1 633 920 *(+3 998)*	28 980 *(+62)*	1 490 037
29.09.2021	1 629 922 *(+3 004)*	28 918 *(+65)*	1 487 800
28.09.2021	1 626 918 *(+2 541)*	28 853 *(+63)*	1 485 862
27.09.2021	1 624 377 *(+3 387)*	28 790 *(+61)*	1 483 976
26.09.2021	1 620 990 *(+3 275)*	28 729 *(+59)*	1 482 416
25.09.2021	1 617 715 *(+3 211)*	28 670 *(+63)*	1 481 203
24.09.2021	1 614 504 *(+2 893)*	28 607 *(+56)*	1 479 958
23.09.2021	1 611 611 *(+3 445)*	28 551 *(+54)*	1 478 681
22.09.2021	1 608 166 *(+1 991)*	28 497 *(+50)*	1 477 350
21.09.2021	1 606 175 *(+1 926)*	28 447 *(+52)*	1 475 997
20.09.2021	1 604 249 *(+2 469)*	28 395 *(+48)*	1 474 697
19.09.2021	1 601 780 *(+2 376)*	28 347 *(+49)*	1 473 474
18.09.2021	1 599 404 *(+2 746)*	28 298 *(+51)*	1 472 259
17.09.2021	1 596 658 *(+2 537)*	28 247 *(+42)*	1 470 979
16.09.2021	1 594 121 *(+2 479)*	28 205 *(+45)*	1 469 620
15.09.2021	1 591 642 *(+1 838)*	28 160 *(+49)*	1 468 151
14.09.2021	1 589 804 *(+1 609)*	28 111 *(+47)*	1 466 721
13.09.2021	1 588 195 *(+2 022)*	28 064 *(+41)*	1 465 359
12.09.2021	1 586 173 *(+1 592)*	28 023 *(+42)*	1 464 100

11.09.2021	1 584 581 *(+1 940)*	27 981 *(+46)*	1 462 853
10.09.2021	1 582 641 *(+1 566)*	27 935 *(+48)*	1 461 536
09.09.2021	1 581 075 *(+1 801)*	27 887 *(+52)*	1 460 093
08.09.2021	1 579 274 *(+1 269)*	27 835 *(+48)*	1 458 632
07.09.2021	1 578 005 *(+1 187)*	27 787 *(+45)*	1 457 119
06.09.2021	1 576 818 *(+1 463)*	27 742 *(+46)*	1 455 678
05.09.2021	1 575 355 *(+1 542)*	27 696 *(+48)*	1 454 400
04.09.2021	1 573 813 *(+1 549)*	27 648 *(+55)*	1 453 242
03.09.2021	1 572 264 *(+1 633)*	27 593 *(+50)*	1 451 886
02.09.2021	1 570 631 *(+1 864)*	27 543 *(+51)*	1 450 330
01.09.2021	1 568 767 *(+1 229)*	27 492 *(+47)*	1 448 644
31.08.2021	1 567 538 *(+1 162)*	27 445 *(+44)*	1 446 708
30.08.2021	1 566 376 *(+1 424)*	27 401 *(+50)*	1 444 794
29.08.2021	1 564 952 *(+1 444)*	27 351 *(+52)*	1 442 955
28.08.2021	1 563 508 *(+1 498)*	27 299 *(+55)*	1 441 166
27.08.2021	1 562 010 *(+1 509)*	27 244 *(+51)*	1 439 126
26.08.2021	1 560 501 *(+1 712)*	27 193 *(+54)*	1 436 829
25.08.2021	1 558 789 *(+1 402)*	27 139 *(+52)*	1 434 366
24.08.2021	1 557 387 *(+1 105)*	27 087 *(+56)*	1 432 067
23.08.2021	1 556 282 *(+1 458)*	27 031 *(+53)*	1 429 919
22.08.2021	1 554 824 *(+1 661)*	26 978 *(+51)*	1 427 913
21.08.2021	1 553 163 *(+1 852)*	26 927 *(+55)*	1 425 773
20.08.2021	1 551 311 *(+1 925)*	26 872 *(+52)*	1 423 229
19.08.2021	1 549 386 *(+2 142)*	26 820 *(+54)*	1 420 630
18.08.2021	1 547 244 *(+1 590)*	26 766 *(+59)*	1 417 825
17.08.2021	1 545 654 *(+2 006)*	26 707 *(+56)*	1 414 796
16.08.2021	1 543 648 *(+1 489)*	26 651 *(+54)*	1 412 329
15.08.2021	1 542 159 *(+1 954)*	26 597 *(+58)*	1 410 177
14.08.2021	1 540 205 *(+2 317)*	26 539 *(+57)*	1 407 723
13.08.2021	1 537 888 *(+2 529)*	26 482 *(+59)*	1 404 879
12.08.2021	1 535 359 *(+2 294)*	26 423 *(+56)*	1 401 836
11.08.2021	1 533 065 *(+2 076)*	26 367 *(+58)*	1 398 507
10.08.2021	1 530 989 *(+1 639)*	26 309 *(+61)*	1 395 285
09.08.2021	1 529 350 *(+2 150)*	26 248 *(+59)*	1 392 213
08.08.2021	1 527 200 *(+2 761)*	26 189 *(+63)*	1 389 595
07.08.2021	1 524 439 *(+2 235)*	26 126 *(+67)*	1 386 543
06.08.2021	1 522 204 *(+2 583)*	26 059 *(+65)*	1 382 967
05.08.2021	1 519 621 *(+3 227)*	25 994 *(+68)*	1 378 924
04.08.2021	1 516 394 *(+2 502)*	25 926 *(+76)*	1 374 447

03.08.2021	1 513 892 *(+1 952)*	25 850 *(+71)*	1 370 145
02.08.2021	1 511 940 *(+3 330)*	25 779 *(+75)*	1 366 386
01.08.2021	1 508 610 *(+2 484)*	25 704 *(+78)*	1 363 505
31.07.2021	1 506 126 *(+3 514)*	25 626 *(+73)*	1 360 030
30.07.2021	1 502 612 *(+3 481)*	25 553 *(+76)*	1 355 871
29.07.2021	1 499 131 *(+3 356)*	25 477 *(+79)*	1 351 297
28.07.2021	1 495 775 *(+2 445)*	25 398 *(+93)*	1 346 237
27.07.2021	1 493 330 *(+2 623)*	25 305 *(+98)*	1 341 106
26.07.2021	1 490 707 *(+2 629)*	25 207 *(+101)*	1 336 194
25.07.2021	1 488 078 *(+3 406)*	25 106 *(+103)*	1 331 614
24.07.2021	1 484 672 *(+3 376)*	25 003 *(+105)*	1 326 132
23.07.2021	1 481 296 *(+3 425)*	24 898 *(+107)*	1 319 555
22.07.2021	1 477 871 *(+4 287)*	24 791 *(+102)*	1 312 545
21.07.2021	1 473 584 *(+3 254)*	24 689 *(+104)*	1 304 958
20.07.2021	1 470 330 *(+3 188)*	24 585 *(+101)*	1 296 952
19.07.2021	1 467 142 *(+4 007)*	24 484 *(+103)*	1 288 984
18.07.2021	1 463 135 *(+4 357)*	24 381 *(+106)*	1 281 319
17.07.2021	1 458 778 *(+4 561)*	24 275 *(+102)*	1 274 036
16.07.2021	1 454 217 *(+5 382)*	24 173 *(+105)*	1 266 393
15.07.2021	1 448 835 *(+5 237)*	24 068 *(+101)*	1 258 361
14.07.2021	1 443 598 *(+3 966)*	23 967 *(+109)*	1 250 506
13.07.2021	1 439 632 *(+4 991)*	23 858 *(+103)*	1 242 987
12.07.2021	1 434 641 *(+5 403)*	23 755 *(+107)*	1 235 697
11.07.2021	1 429 238 *(+5 410)*	23 648 *(+110)*	1 228 601
10.07.2021	1 423 828 *(+5 694)*	23 538 *(+108)*	1 221 766
09.07.2021	1 418 134 *(+6 643)*	23 430 *(+102)*	1 214 731
08.07.2021	1 411 491 *(+6 040)*	23 328 *(+109)*	1 207 500
07.07.2021	1 405 451 *(+5 621)*	23 219 *(+104)*	1 200 695
06.07.2021	1 399 830 *(+5 498)*	23 115 *(+114)*	1 194 207
05.07.2021	1 394 332 *(+6 557)*	23 001 *(+106)*	1 188 188
04.07.2021	1 387 775 *(+7 624)*	22 895 *(+111)*	1 182 540
03.07.2021	1 380 151 *(+7 446)*	22 784 *(+116)*	1 176 793
02.07.2021	1 372 705 *(+6 893)*	22 668 *(+112)*	1 170 924
01.07.2021	1 365 812 *(+7 597)*	22 556 *(+108)*	1 164 653
30.06.2021	1 358 215 *(+5 823)*	22 448 *(+117)*	1 158 719
29.06.2021	1 352 392 *(+6 209)*	22 331 *(+121)*	1 153 145
28.06.2021	1 346 183 *(+7 246)*	22 210 *(+124)*	1 148 291
27.06.2021	1 338 937 *(+6 723)*	22 086 *(+114)*	1 143 858
26.06.2021	1 332 214 *(+8 457)*	21 972 *(+96)*	1 139 840

25.06.2021	1 323 757 *(+7 916)*	21 876 *(+98)*	1 135 580
24.06.2021	1 315 841 *(+8 598)*	21 778 *(+92)*	1 131 022
23.06.2021	1 307 243 *(+6 534)*	21 686 *(+88)*	1 126 965
22.06.2021	1 300 709 *(+6 555)*	21 598 *(+86)*	1 123 295
21.06.2021	1 294 154 *(+7 584)*	21 512 *(+79)*	1 119 837
20.06.2021	1 286 570 *(+8 305)*	21 433 *(+81)*	1 116 739
19.06.2021	1 278 265 *(+9 120)*	21 352 *(+76)*	1 113 794
18.06.2021	1 269 145 *(+9 056)*	21 276 *(+78)*	1 110 399
17.06.2021	1 260 089 *(+6 195)*	21 198 *(+73)*	1 106 615
16.06.2021	1 253 894 *(+5 782)*	21 125 *(+75)*	1 102 979
15.06.2021	1 248 112 *(+6 805)*	21 050 *(+71)*	1 099 598
14.06.2021	1 241 307 *(+6 590)*	20 979 *(+72)*	1 096 553
13.06.2021	1 234 717 *(+7 704)*	20 907 *(+69)*	1 093 738
12.06.2021	1 227 013 *(+6 701)*	20 838 *(+67)*	1 090 700
11.06.2021	1 220 312 *(+5 853)*	20 771 *(+60)*	1 087 975
10.06.2021	1 214 459 *(+5 245)*	20 711 *(+63)*	1 084 914
09.06.2021	1 209 214 *(+4 124)*	20 648 *(+58)*	1 082 243
08.06.2021	1 205 090 *(+3 817)*	20 590 *(+62)*	1 079 932
07.06.2021	1 201 273 *(+3 266)*	20 528 *(+59)*	1 077 914
06.06.2021	1 198 007 *(+2 936)*	20 469 *(+61)*	1 076 346
05.06.2021	1 195 071 *(+2 897)*	20 408 *(+56)*	1 074 605
04.06.2021	1 192 174 *(+2 817)*	20 352 *(+58)*	1 072 425
03.06.2021	1 189 357 *(+2 876)*	20 294 *(+57)*	1 070 107
02.06.2021	1 186 481 *(+2 842)*	20 237 *(+60)*	1 067 425
01.06.2021	1 183 639 *(+3 669)*	20 177 *(+62)*	1 064 858
31.05.2021	1 179 970 *(+2 614)*	20 115 *(+55)*	1 062 465
30.05.2021	1 177 356 *(+3 719)*	20 060 *(+59)*	1 060 588
29.05.2021	1 173 637 *(+3 241)*	20 001 *(+61)*	1 058 041
28.05.2021	1 170 396 *(+3 274)*	19 940 *(+56)*	1 055 130
27.05.2021	1 167 122 *(+3 105)*	19 884 *(+58)*	1 052 132
26.05.2021	1 164 017 *(+2 416)*	19 826 *(+62)*	1 049 246
25.05.2021	1 161 601 *(+2 075)*	19 764 *(+57)*	1 046 726
24.05.2021	1 159 526 *(+2 487)*	19 707 *(+59)*	1 044 518
23.05.2021	1 157 039 *(+2 924)*	19 648 *(+56)*	1 042 788
22.05.2021	1 154 115 *(+2 653)*	19 592 *(+60)*	1 040 760
21.05.2021	1 151 462 *(+2 954)*	19 532 *(+58)*	1 038 332
20.05.2021	1 148 508 *(+3 312)*	19 474 *(+55)*	1 035 739
19.05.2021	1 145 196 *(+2 096)*	19 419 *(+61)*	1 032 862
18.05.2021	1 143 100 *(+2 430)*	19 358 *(+59)*	1 030 199

17.05.2021	1 140 670 *(+3 573)*	19 299 *(+57)*	1 027 835
16.05.2021	1 137 097 *(+2 789)*	19 242 *(+56)*	1 025 794
15.05.2021	1 134 308 *(+3 073)*	19 186 *(+58)*	1 023 380
14.05.2021	1 131 235 *(+3 818)*	19 128 *(+60)*	1 020 683
13.05.2021	1 127 417 *(+2 787)*	19 068 *(+55)*	1 017 740
12.05.2021	1 124 630 *(+2 718)*	19 013 *(+59)*	1 015 097
11.05.2021	1 121 912 *(+2 628)*	18 954 *(+61)*	1 012 703
10.05.2021	1 119 284 *(+2 896)*	18 893 *(+56)*	1 010 652
09.05.2021	1 116 388 *(+2 828)*	18 837 *(+58)*	1 008 676
08.05.2021	1 113 560 *(+2 765)*	18 779 *(+57)*	1 006 542
07.05.2021	1 110 795 *(+2 846)*	18 722 *(+60)*	1 004 060
06.05.2021	1 107 949 *(+2 114)*	18 662 *(+59)*	1 001 798
05.05.2021	1 105 835 *(+2 432)*	18 603 *(+56)*	999 742
04.05.2021	1 103 403 *(+2 050)*	18 547 *(+61)*	997 957
03.05.2021	1 101 353 *(+2 635)*	18 486 *(+58)*	996 373
02.05.2021	1 098 718 *(+2 699)*	18 428 *(+54)*	994 965
01.05.2021	1 096 019 *(+3 208)*	18 374 *(+59)*	993 348
30.04.2021	1 092 811 *(+2 662)*	18 315 *(+57)*	991 419
29.04.2021	1 090 149 *(+3 215)*	18 258 *(+56)*	989 411
28.04.2021	1 086 934 *(+1 840)*	18 202 *(+54)*	987 457
27.04.2021	1 085 094 *(+2 098)*	18 148 *(+58)*	985 769
26.04.2021	1 082 996 *(+2 687)*	18 090 *(+55)*	984 346
25.04.2021	1 080 309 *(+2 526)*	18 035 *(+53)*	983 221
24.04.2021	1 077 783 *(+2 541)*	17 982 *(+59)*	981 882
23.04.2021	1 075 242 *(+2 502)*	17 923 *(+57)*	980 003
22.04.2021	1 072 740 *(+2 704)*	17 866 *(+54)*	977 880
21.04.2021	1 070 036 *(+1 988)*	17 812 *(+58)*	975 973
20.04.2021	1 068 048 *(+1 996)*	17 754 *(+56)*	974 229
19.04.2021	1 066 052 *(+2 279)*	17 698 *(+59)*	972 646
18.04.2021	1 063 773 *(+2 252)*	17 639 *(+53)*	971 557
17.04.2021	1 061 521 *(+2 822)*	17 586 *(+58)*	970 320
16.04.2021	1 058 699 *(+2 476)*	17 528 *(+57)*	968 682
15.04.2021	1 056 223 *(+2 455)*	17 471 *(+59)*	966 781
14.04.2021	1 053 768 *(+1 837)*	17 412 *(+52)*	964 837
13.04.2021	1 051 931 *(+1 782)*	17 360 *(+58)*	963 086
12.04.2021	1 050 149 *(+1 833)*	17 302 *(+54)*	961 392
11.04.2021	1 048 316 *(+2 090)*	17 248 *(+56)*	960 171
10.04.2021	1 046 226 *(+2 006)*	17 192 *(+53)*	958 783
09.04.2021	1 044 220 *(+2 421)*	17 139 *(+57)*	957 137

08.04.2021	1 041 799 *(+2 024)*	17 082 *(+55)*	955 148
07.04.2021	1 039 775 *(+1 585)*	17 027 *(+51)*	953 402
06.04.2021	1 038 190 *(+1 747)*	16 976 *(+54)*	951 851
05.04.2021	1 036 443 *(+1 876)*	16 922 *(+50)*	950 658
04.04.2021	1 034 567 *(+1 901)*	16 872 *(+56)*	949 843
03.04.2021	1 032 666 *(+2 011)*	16 816 *(+52)*	948 845
02.04.2021	1 030 655 *(+1 764)*	16 764 *(+57)*	947 694
01.04.2021	1 028 891 *(+2 151)*	16 707 *(+51)*	946 370
31.03.2021	1 026 740 *(+1 286)*	16 656 *(+53)*	944 738
30.03.2021	1 025 454 *(+1 291)*	16 603 *(+56)*	943 201
29.03.2021	1 024 163 *(+1 612)*	16 547 *(+49)*	942 036
28.03.2021	1 022 551 *(+1 878)*	16 498 *(+52)*	941 192
27.03.2021	1 020 673 *(+1 551)*	16 446 *(+54)*	940 041
26.03.2021	1 019 122 *(+1 813)*	16 392 *(+55)*	938 673
25.03.2021	1 017 309 *(+1 787)*	16 337 *(+49)*	937 122
24.03.2021	1 015 522 *(+1 431)*	16 288 *(+53)*	935 400
23.03.2021	1 014 091 *(+1 042)*	16 235 *(+51)*	933 807
22.03.2021	1 013 049 *(+1 586)*	16 184 *(+56)*	932 428
21.03.2021	1 011 463 *(+1 511)*	16 128 *(+50)*	931 296
20.03.2021	1 009 952 *(+1 728)*	16 078 *(+54)*	929 925
19.03.2021	1 008 224 *(+1 809)*	16 024 *(+51)*	928 156
18.03.2021	1 006 415 *(+1 934)*	15 973 *(+48)*	926 254
17.03.2021	1 004 481 *(+1 201)*	15 925 *(+53)*	924 494
16.03.2021	1 003 280 *(+1 533)*	15 872 *(+57)*	922 899
15.03.2021	1 001 747 *(+1 353)*	15 815 *(+51)*	921 424
14.03.2021	1 000 394 *(+1 905)*	15 764 *(+55)*	920 388
13.03.2021	998 489 *(+1 600)*	15 709 *(+52)*	919 278
12.03.2021	996 889 *(+1 610)*	15 657 *(+56)*	917 809
11.03.2021	995 279 *(+1 281)*	15 601 *(+54)*	916 204
10.03.2021	993 998 *(+1 116)*	15 547 *(+51)*	914 626
09.03.2021	992 882 *(+1 066)*	15 496 *(+49)*	913 173
08.03.2021	991 816 *(+1 421)*	15 447 *(+53)*	912 146
07.03.2021	990 395 *(+1 534)*	15 394 *(+47)*	911 109
06.03.2021	988 861 *(+1 820)*	15 347 *(+50)*	909 692
05.03.2021	987 041 *(+1 757)*	15 297 *(+54)*	907 770
04.03.2021	985 284 *(+2 150)*	15 243 *(+51)*	905 521
03.03.2021	983 134 *(+1 284)*	15 192 *(+46)*	903 100
02.03.2021	981 850 *(+1 277)*	15 146 *(+49)*	901 035
01.03.2021	980 573 *(+2 097)*	15 097 *(+42)*	899 239

28.02.2021	978 476 *(+1 737)*	15 055 *(+48)*	897 698
27.02.2021	976 739 *(+1 825)*	15 007 *(+41)*	895 879
26.02.2021	974 914 *(+1 336)*	14 966 *(+45)*	893 871
25.02.2021	973 578 *(+1 406)*	14 921 *(+47)*	891 448
24.02.2021	972 172 *(+1 417)*	14 874 *(+43)*	889 356
23.02.2021	970 755 *(+1 198)*	14 831 *(+49)*	887 637
22.02.2021	969 557 *(+1 723)*	14 782 *(+41)*	886 273
21.02.2021	967 834 *(+1 602)*	14 741 *(+52)*	884 615
20.02.2021	966 232 *(+1 623)*	14 689 *(+58)*	882 808
19.02.2021	964 609 *(+1 972)*	14 631 *(+53)*	880 767
18.02.2021	962 637 *(+1 950)*	14 578 *(+55)*	878 655
17.02.2021	960 687 *(+1 282)*	14 523 *(+59)*	876 141
16.02.2021	959 405 *(+1 409)*	14 464 *(+60)*	873 898
15.02.2021	957 996 *(+1 818)*	14 404 *(+56)*	871 836
14.02.2021	956 178 *(+1 559)*	14 348 *(+58)*	869 869
13.02.2021	954 619 *(+1 963)*	14 290 *(+67)*	867 780
12.02.2021	952 656 *(+2 139)*	14 223 *(+63)*	865 100
11.02.2021	950 517 *(+2 040)*	14 160 *(+61)*	861 916
10.02.2021	948 477 *(+1 441)*	14 099 *(+68)*	858 358
09.02.2021	947 036 *(+1 584)*	14 031 *(+73)*	854 725
08.02.2021	945 452 *(+1 728)*	13 958 *(+62)*	851 171
07.02.2021	943 724 *(+2 028)*	13 896 *(+64)*	847 822
06.02.2021	941 696 *(+2 214)*	13 832 *(+60)*	844 165
05.02.2021	939 482 *(+2 032)*	13 772 *(+71)*	839 832
04.02.2021	937 450 *(+2 095)*	13 701 *(+67)*	835 168
03.02.2021	935 355 *(+1 545)*	13 634 *(+65)*	829 844
02.02.2021	933 810 *(+1 701)*	13 569 *(+74)*	824 623
01.02.2021	932 109 *(+2 037)*	13 495 *(+61)*	819 611
31.01.2021	930 072 *(+2 284)*	13 434 *(+66)*	814 728
30.01.2021	927 788 *(+2 430)*	13 368 *(+68)*	809 226
29.01.2021	925 358 *(+2 799)*	13 300 *(+63)*	803 642
28.01.2021	922 559 *(+2 897)*	13 237 *(+62)*	797 462
27.01.2021	919 662 *(+1 837)*	13 175 *(+75)*	791 320
26.01.2021	917 825 *(+2 317)*	13 100 *(+70)*	785 396
25.01.2021	915 508 *(+2 382)*	13 030 *(+61)*	779 824
24.01.2021	913 126 *(+3 069)*	12 969 *(+74)*	774 550
23.01.2021	910 057 *(+2 668)*	12 895 *(+64)*	769 433
22.01.2021	907 389 *(+3 037)*	12 831 *(+72)*	763 500
21.01.2021	904 352 *(+3 458)*	12 759 *(+69)*	757 871

20.01.2021	900 894 *(+2 452)*	12 690 *(+65)*	752 651
19.01.2021	898 442 *(+3 115)*	12 625 *(+84)*	747 881
18.01.2021	895 327 *(+3 679)*	12 541 *(+73)*	743 349
17.01.2021	891 648 *(+4 012)*	12 468 *(+67)*	739 340
16.01.2021	887 636 *(+4 674)*	12 401 *(+79)*	734 728
15.01.2021	882 962 *(+5 534)*	12 322 *(+81)*	729 089
14.01.2021	877 428 *(+5 893)*	12 241 *(+68)*	722 487
13.01.2021	871 535 *(+4 320)*	12 173 *(+76)*	715 438
12.01.2021	867 215 *(+5 001)*	12 097 *(+72)*	708 568
11.01.2021	862 214 *(+4 646)*	12 025 *(+75)*	701 790
10.01.2021	857 568 *(+4 216)*	11 950 *(+77)*	695 106
09.01.2021	853 352 *(+4 900)*	11 873 *(+74)*	688 061
08.01.2021	848 452 *(+5 027)*	11 799 *(+71)*	680 759
07.01.2021	843 425 *(+4 871)*	11 728 *(+76)*	673 692
06.01.2021	838 554 *(+5 142)*	11 652 *(+70)*	666 282
05.01.2021	833 412 *(+4 842)*	11 582 *(+75)*	659 076
04.01.2021	828 570 *(+3 591)*	11 507 *(+74)*	652 324
03.01.2021	824 979 *(+3 851)*	11 433 *(+77)*	645 923
02.01.2021	821 128 *(+5 452)*	11 356 *(+76)*	639 809
01.01.2021	815 676 *(+5 907)*	11 280 *(+71)*	633 702
31.12.2020	809 769 *(+6 566)*	11 209 *(+73)*	627 368
30.12.2020	803 203 *(+5 105)*	11 136 *(+72)*	621 292
29.12.2020	798 098 *(+5 641)*	11 064 *(+75)*	615 396
28.12.2020	792 457 *(+6 253)*	10 989 *(+76)*	609 770
27.12.2020	786 204 *(+6 620)*	10 913 *(+73)*	604 604
26.12.2020	779 584 *(+7 480)*	10 840 *(+77)*	599 541
25.12.2020	772 104 *(+7 315)*	10 763 *(+74)*	593 939
24.12.2020	764 789 *(+8 203)*	10 689 *(+76)*	588 280
23.12.2020	756 586 *(+5 652)*	10 613 *(+73)*	582 772
22.12.2020	750 934 *(+7 237)*	10 540 *(+71)*	577 544
21.12.2020	743 697 *(+7 797)*	10 469 *(+75)*	572 541
20.12.2020	735 900 *(+7 263)*	10 394 *(+77)*	567 743
19.12.2020	728 637 *(+6 459)*	10 317 *(+74)*	562 953
18.12.2020	722 178 *(+6 937)*	10 243 *(+72)*	557 028
17.12.2020	715 241 *(+6 711)*	10 171 *(+76)*	551 207
16.12.2020	708 530 *(+5 028)*	10 095 *(+73)*	545 430
15.12.2020	703 502 *(+5 418)*	10 022 *(+77)*	539 859
14.12.2020	698 084 *(+5 874)*	9 945 *(+75)*	534 552
13.12.2020	692 210 *(+6 425)*	9 870 *(+72)*	529 620

12.12.2020	685 785 *(+6 622)*	9 798 *(+76)*	524 779
11.12.2020	679 163 *(+7 215)*	9 722 *(+77)*	518 889
10.12.2020	671 948 *(+6 730)*	9 645 *(+74)*	512 969
09.12.2020	665 218 *(+5 145)*	9 571 *(+75)*	507 149
08.12.2020	660 073 *(+5 232)*	9 496 *(+71)*	501 403
07.12.2020	654 841 *(+7 279)*	9 425 *(+76)*	495 871
06.12.2020	647 562 *(+7 512)*	9 349 *(+72)*	490 844
05.12.2020	640 050 *(+7 993)*	9 277 *(+74)*	485 308
04.12.2020	632 057 *(+6 868)*	9 203 *(+77)*	478 724
03.12.2020	625 189 *(+7 750)*	9 126 *(+73)*	471 833
02.12.2020	617 439 *(+5 191)*	9 053 *(+75)*	464 984
01.12.2020	612 248 *(+6 524)*	8 978 *(+76)*	458 409
30.11.2020	605 724 *(+6 511)*	8 902 *(+74)*	452 184
29.11.2020	599 213 *(+6 798)*	8 828 *(+72)*	447 111
28.11.2020	592 415 *(+7 320)*	8 756 *(+76)*	441 535
27.11.2020	585 095 *(+7 918)*	8 680 *(+77)*	434 902
26.11.2020	577 177 *(+6 075)*	8 603 *(+73)*	428 418
25.11.2020	571 102 *(+4 685)*	8 530 *(+75)*	422 189
24.11.2020	566 417 *(+5 838)*	8 455 *(+76)*	416 242
23.11.2020	560 579 *(+6 866)*	8 379 *(+71)*	410 641
22.11.2020	553 713 *(+6 575)*	8 308 *(+75)*	405 577
21.11.2020	547 138 *(+7 168)*	8 233 *(+74)*	400 332
20.11.2020	539 970 *(+6 902)*	8 159 *(+77)*	394 252
19.11.2020	533 068 *(+6 438)*	8 082 *(+73)*	388 416
18.11.2020	526 630 *(+4 174)*	8 009 *(+76)*	382 634
17.11.2020	522 456 *(+5 882)*	7 933 *(+74)*	377 131
16.11.2020	516 574 *(+6 360)*	7 859 *(+72)*	371 857
15.11.2020	510 214 *(+6 271)*	7 787 *(+75)*	367 613
14.11.2020	503 943 *(+6 427)*	7 712 *(+69)*	363 347
13.11.2020	497 516 *(+5 974)*	7 643 *(+70)*	358 924
12.11.2020	491 542 *(+5 997)*	7 573 *(+71)*	354 705
11.11.2020	485 545 *(+4 477)*	7 502 *(+73)*	350 746
10.11.2020	481 068 *(+5 902)*	7 429 *(+68)*	346 889
09.11.2020	475 166 *(+6 897)*	7 361 *(+72)*	343 226
08.11.2020	468 269 *(+5 751)*	7 289 *(+74)*	339 913
07.11.2020	462 518 *(+5 829)*	7 215 *(+75)*	336 659
06.11.2020	456 689 *(+6 253)*	7 140 *(+69)*	332 984
05.11.2020	450 436 *(+5 255)*	7 071 *(+67)*	329 201
04.11.2020	445 181 *(+5 826)*	7 004 *(+68)*	325 531

03.11.2020	439 355 *(+5 150)*	6 936 *(+63)*	321 679
02.11.2020	434 205 *(+4 796)*	6 873 *(+53)*	318 032
01.11.2020	429 409 *(+5 261)*	6 820 *(+52)*	314 791
31.10.2020	424 148 *(+4 952)*	6 768 *(+55)*	311 450
30.10.2020	419 196 *(+5 268)*	6 713 *(+69)*	307 816
29.10.2020	413 928 *(+4 906)*	6 644 *(+66)*	303 831
28.10.2020	409 022 *(+3 670)*	6 578 *(+75)*	300 051
27.10.2020	405 352 *(+4 312)*	6 503 *(+61)*	296 474
26.10.2020	401 040 *(+5 224)*	6 442 *(+62)*	293 205
25.10.2020	395 816 *(+4 455)*	6 380 *(+68)*	290 524
24.10.2020	391 361 *(+4 453)*	6 312 *(+63)*	287 910
23.10.2020	386 908 *(+5 478)*	6 249 *(+62)*	285 271
22.10.2020	381 430 *(+4 413)*	6 187 *(+66)*	282 492
21.10.2020	377 017 *(+4 389)*	6 121 *(+63)*	279 973
20.10.2020	372 628 *(+4 999)*	6 058 *(+49)*	277 608
19.10.2020	367 629 *(+5 376)*	6 009 *(+51)*	275 648
18.10.2020	362 253 *(+4 610)*	5 958 *(+52)*	273 881
17.10.2020	357 643 *(+4 648)*	5 906 *(+56)*	272 227
16.10.2020	352 995 *(+5 049)*	5 850 *(+54)*	270 226
15.10.2020	347 946 *(+3 942)*	5 796 *(+57)*	268 423
14.10.2020	344 004 *(+4 573)*	5 739 *(+52)*	266 470
13.10.2020	339 431 *(+4 618)*	5 687 *(+58)*	264 500
12.10.2020	334 813 *(+4 395)*	5 629 *(+34)*	262 750
11.10.2020	330 418 *(+4 501)*	5 595 *(+35)*	261 619
10.10.2020	325 917 *(+4 105)*	5 560 *(+30)*	260 433
09.10.2020	321 812 *(+3 701)*	5 530 *(+33)*	259 127
08.10.2020	318 111 *(+3 323)*	5 497 *(+55)*	257 703
07.10.2020	314 788 *(+3 229)*	5 442 *(+41)*	256 362
06.10.2020	311 559 *(+4 082)*	5 401 *(+31)*	255 100
05.10.2020	307 477 *(+3 537)*	5 370 *(+27)*	253 944
04.10.2020	303 940 *(+3 327)*	5 343 *(+29)*	253 031
03.10.2020	300 613 *(+2 884)*	5 314 *(+32)*	252 087
02.10.2020	297 729 *(+2 704)*	5 282 *(+28)*	251 018
01.10.2020	295 025 *(+2 424)*	5 254 *(+24)*	249 861
30.09.2020	292 601 *(+2 308)*	5 230 *(+27)*	248 650
29.09.2020	290 293 *(+2 300)*	5 203 *(+23)*	247 301
28.09.2020	287 993 *(+2 217)*	5 180 *(+16)*	246 074
27.09.2020	285 776 *(+2 016)*	5 164 *(+18)*	245 132
26.09.2020	283 760 *(+1 792)*	5 146 *(+17)*	244 006

25.09.2020	281 968 *(+1 560)*	5 129 *(+14)*	242 883
24.09.2020	280 408 *(+1 050)*	5 115 *(+15)*	241 656
23.09.2020	279 358 *(+970)*	5 100 *(+16)*	240 352
22.09.2020	278 388 *(+980)*	5 084 *(+15)*	239 126
21.09.2020	277 408 *(+915)*	5 069 *(+12)*	237 935
20.09.2020	276 493 *(+860)*	5 057 *(+13)*	236 947
19.09.2020	275 633 *(+825)*	5 044 *(+11)*	236 055
18.09.2020	274 808 *(+805)*	5 033 *(+8)*	235 136
17.09.2020	274 003 *(+730)*	5 025 *(+9)*	234 029
16.09.2020	273 273 *(+750)*	5 016 *(+10)*	232 706
15.09.2020	272 523 *(+730)*	5 006 *(+13)*	231 483
14.09.2020	271 793 *(+696)*	4 993 *(+11)*	230 397
13.09.2020	271 097 *(+650)*	4 982 *(+14)*	229 573
12.09.2020	270 447 *(+670)*	4 968 *(+12)*	228 673
11.09.2020	269 777 *(+698)*	4 956 *(+9)*	227 530
10.09.2020	269 079 *(+695)*	4 947 *(+14)*	226 274
09.09.2020	268 384 *(+642)*	4 933 *(+12)*	224 840
08.09.2020	267 742 *(+695)*	4 921 *(+16)*	223 502
07.09.2020	267 047 *(+690)*	4 905 *(+14)*	222 252
06.09.2020	266 357 *(+620)*	4 891 *(+13)*	221 370
05.09.2020	265 737 *(+671)*	4 878 *(+11)*	220 407
04.09.2020	265 066 *(+692)*	4 867 *(+10)*	219 095
03.09.2020	264 374 *(+690)*	4 857 *(+13)*	217 899
02.09.2020	263 684 *(+625)*	4 844 *(+12)*	216 742
01.09.2020	263 059 *(+641)*	4 832 *(+11)*	215 383
31.08.2020	262 418 *(+685)*	4 821 *(+12)*	214 121
30.08.2020	261 733 *(+695)*	4 809 *(+11)*	213 205
29.08.2020	261 038 *(+677)*	4 798 *(+12)*	212 301
28.08.2020	260 361 *(+654)*	4 786 *(+10)*	210 908
27.08.2020	259 707 *(+637)*	4 776 *(+12)*	209 628
26.08.2020	259 070 *(+640)*	4 764 *(+11)*	208 193
25.08.2020	258 430 *(+681)*	4 753 *(+12)*	206 804
24.08.2020	257 749 *(+625)*	4 741 *(+10)*	205 649
23.08.2020	257 124 *(+611)*	4 731 *(+11)*	204 786
22.08.2020	256 513 *(+687)*	4 720 *(+10)*	203 821
21.08.2020	255 826 *(+690)*	4 710 *(+12)*	202 727
20.08.2020	255 136 *(+688)*	4 698 *(+11)*	201 567
19.08.2020	254 448 *(+691)*	4 687 *(+10)*	200 359
18.08.2020	253 757 *(+693)*	4 677 *(+11)*	199 117

17.08.2020	253 064 *(+690)*	4 666 *(+10)*	197 962
16.08.2020	252 374 *(+688)*	4 656 *(+11)*	197 143
15.08.2020	251 686 *(+695)*	4 645 *(+12)*	196 098
14.08.2020	250 991 *(+688)*	4 633 *(+11)*	194 823
13.08.2020	250 303 *(+692)*	4 622 *(+11)*	193 473
12.08.2020	249 611 *(+689)*	4 611 *(+12)*	192 686
11.08.2020	248 922 *(+694)*	4 599 *(+14)*	191 598
10.08.2020	248 228 *(+694)*	4 585 *(+13)*	190 555
09.08.2020	247 534 *(+689)*	4 572 *(+12)*	189 657
08.08.2020	246 845 *(+691)*	4 560 *(+14)*	188 854
07.08.2020	246 154 *(+686)*	4 546 *(+12)*	187 576
06.08.2020	245 468 *(+684)*	4 534 *(+13)*	186 223
05.08.2020	244 784 *(+687)*	4 521 *(+11)*	184 969
04.08.2020	244 097 *(+691)*	4 510 *(+12)*	183 640
03.08.2020	243 406 *(+693)*	4 498 *(+13)*	182 184
02.08.2020	242 713 *(+664)*	4 485 *(+12)*	181 342
01.08.2020	242 049 *(+690)*	4 473 *(+13)*	180 581
31.07.2020	241 359 *(+695)*	4 460 *(+14)*	179 168
30.07.2020	240 664 *(+678)*	4 446 *(+12)*	177 908
29.07.2020	239 986 *(+671)*	4 434 *(+13)*	176 437
28.07.2020	239 315 *(+674)*	4 421 *(+10)*	175 812
27.07.2020	238 641 *(+694)*	4 411 *(+13)*	174 985
26.07.2020	237 947 *(+683)*	4 398 *(+9)*	174 721
25.07.2020	237 264 *(+648)*	4 389 *(+14)*	174 415
24.07.2020	236 616 *(+645)*	4 375 *(+11)*	173 854
23.07.2020	235 971 *(+608)*	4 364 *(+14)*	173 392
22.07.2020	235 363 *(+638)*	4 350 *(+19)*	172 939
21.07.2020	234 725 *(+602)*	4 331 *(+17)*	171 771
20.07.2020	234 123 *(+578)*	4 314 *(+15)*	170 918
19.07.2020	233 545 *(+591)*	4 299 *(+14)*	170 539
18.07.2020	232 954 *(+578)*	4 285 *(+14)*	170 180
17.07.2020	232 376 *(+575)*	4 271 *(+13)*	169 637
16.07.2020	231 801 *(+531)*	4 258 *(+24)*	168 955
15.07.2020	231 270 *(+628)*	4 234 *(+29)*	167 810
14.07.2020	230 642 *(+613)*	4 205 *(+37)*	165 966
13.07.2020	230 029 *(+672)*	4 168 *(+25)*	164 954
12.07.2020	229 357 *(+679)*	4 143 *(+27)*	164 560
11.07.2020	228 678 *(+678)*	4 116 *(+29)*	164 095
10.07.2020	228 000 *(+637)*	4 087 *(+28)*	163 093

09.07.2020	227 363 *(+568)*	4 059 *(+32)*	162 086
08.07.2020	226 795 *(+621)*	4 027 *(+28)*	160 913
07.07.2020	226 174 *(+629)*	3 999 *(+24)*	159 718
06.07.2020	225 545 *(+685)*	3 975 *(+22)*	157 652
05.07.2020	224 860 *(+650)*	3 953 *(+24)*	157 020
04.07.2020	224 210 *(+680)*	3 929 *(+25)*	156 459
03.07.2020	223 530 *(+659)*	3 904 *(+34)*	154 839
02.07.2020	222 871 *(+662)*	3 870 *(+39)*	153 248
01.07.2020	222 209 *(+611)*	3 831 *(+35)*	152 193
30.06.2020	221 598 *(+745)*	3 796 *(+35)*	150 397
29.06.2020	220 853 *(+782)*	3 761 *(+23)*	148 102
28.06.2020	220 071 *(+717)*	3 738 *(+24)*	147 095
27.06.2020	219 354 *(+750)*	3 714 *(+20)*	146 043
26.06.2020	218 604 *(+813)*	3 694 *(+25)*	144 191
25.06.2020	217 791 *(+885)*	3 669 *(+12)*	142 194
24.06.2020	216 906 *(+811)*	3 657 *(+14)*	141 268
23.06.2020	216 095 *(+1 081)*	3 643 *(+26)*	139 248
22.06.2020	215 014 *(+1 068)*	3 617 *(+20)*	136 670
21.06.2020	213 946 *(+968)*	3 597 *(+32)*	135 556
20.06.2020	212 978 *(+1 057)*	3 565 *(+34)*	134 549
19.06.2020	211 921 *(+1 136)*	3 531 *(+48)*	132 434
18.06.2020	210 785 *(+1 040)*	3 483 *(+49)*	130 431
17.06.2020	209 745 *(+1 065)*	3 434 *(+48)*	128 385
16.06.2020	208 680 *(+1 416)*	3 386 *(+52)*	126 114
15.06.2020	207 264 *(+1 359)*	3 334 *(+53)*	123 240
14.06.2020	205 905 *(+1 477)*	3 281 *(+50)*	121 691
13.06.2020	204 428 *(+1 493)*	3 231 *(+44)*	119 558
12.06.2020	202 935 *(+1 714)*	3 187 *(+49)*	118 024
11.06.2020	201 221 *(+1 436)*	3 138 *(+53)*	115 769
10.06.2020	199 785 *(+1 195)*	3 085 *(+56)*	113 533
09.06.2020	198 590 *(+1 572)*	3 029 *(+59)*	109 737
08.06.2020	197 018 *(+2 001)*	2 970 *(+51)*	104 347
07.06.2020	195 017 *(+1 956)*	2 919 *(+55)*	102 714
06.06.2020	193 061 *(+1 992)*	2 864 *(+58)*	100 431
05.06.2020	191 069 *(+1 855)*	2 806 *(+57)*	97 358
04.06.2020	189 214 *(+1 998)*	2 749 *(+64)*	94 715
03.06.2020	187 216 *(+1 842)*	2 685 *(+61)*	91 654
02.06.2020	185 374 *(+2 286)*	2 624 *(+71)*	88 269
01.06.2020	183 088 *(+2 297)*	2 553 *(+76)*	82 239

31.05.2020	180 791 *(+2 595)*	2 477 *(+69)*	80 179
30.05.2020	178 196 *(+2 367)*	2 408 *(+78)*	78 324
29.05.2020	175 829 *(+2 332)*	2 330 *(+76)*	74 725
28.05.2020	173 497 *(+2 054)*	2 254 *(+71)*	71 251
27.05.2020	171 443 *(+2 140)*	2 183 *(+73)*	67 458
26.05.2020	169 303 *(+2 830)*	2 110 *(+76)*	61 619
25.05.2020	166 473 *(+2 560)*	2 034 *(+41)*	53 586
24.05.2020	163 913 *(+2 516)*	1 993 *(+59)*	49 840
23.05.2020	161 397 *(+3 190)*	1 934 *(+67)*	47 413
22.05.2020	158 207 *(+2 988)*	1 867 *(+73)*	43 582
21.05.2020	155 219 *(+2 913)*	1 794 *(+68)*	40 682
20.05.2020	152 306 *(+2 699)*	1 726 *(+75)*	36 936
19.05.2020	149 607 *(+3 545)*	1 651 *(+71)*	31 496
18.05.2020	146 062 *(+3 238)*	1 580 *(+77)*	28 913
17.05.2020	142 824 *(+3 855)*	1 503 *(+71)*	27 490
16.05.2020	138 969 *(+3 505)*	1 432 *(+74)*	26 032
15.05.2020	135 464 *(+4 748)*	1 358 *(+68)*	24 562
14.05.2020	130 716 *(+4 712)*	1 290 *(+58)*	23 327
13.05.2020	126 004 *(+4 703)*	1 232 *(+53)*	21 506
12.05.2020	121 301 *(+5 392)*	1 179 *(+55)*	19 642
11.05.2020	115 909 *(+6 169)*	1 124 *(+56)*	17 822
10.05.2020	109 740 *(+5 551)*	1 068 *(+58)*	13 790
09.05.2020	104 189 *(+5 667)*	1 010 *(+54)*	12 779
08.05.2020	98 522 *(+5 846)*	956 *(+51)*	10 259
07.05.2020	92 676 *(+6 703)*	905 *(+39)*	9 227
06.05.2020	85 973 *(+5 858)*	866 *(+50)*	8 458
05.05.2020	80 115 *(+5 714)*	816 *(+52)*	7 870
04.05.2020	74 401 *(+5 795)*	764 *(+35)*	7 573
03.05.2020	68 606 *(+5 948)*	729 *(+34)*	7 029
02.05.2020	62 658 *(+5 358)*	695 *(+37)*	6 374
01.05.2020	57 300 *(+3 561)*	658 *(+47)*	5 766
30.04.2020	53 739 *(+3 093)*	611 *(+65)*	5 135
29.04.2020	50 646 *(+2 220)*	546 *(+67)*	4 610
28.04.2020	48 426 *(+3 075)*	479 *(+44)*	4 130
27.04.2020	45 351 *(+2 871)*	435 *(+31)*	3 524
26.04.2020	42 480 *(+2 971)*	404 *(+38)*	3 175
25.04.2020	39 509 *(+2 612)*	366 *(+41)*	3 047
24.04.2020	36 897 *(+2 957)*	325 *(+37)*	2 735
23.04.2020	33 940 *(+1 959)*	288 *(+27)*	2 448

22.04.2020	31 981 (+2 548)	261 (+28)	2 267
21.04.2020	29 433 (+3 083)	233 (+29)	2 057
20.04.2020	26 350 (+2 026)	204 (+28)	1 838
19.04.2020	24 324 (+3 570)	176 (+28)	1 763
18.04.2020	20 754 (+2 649)	148 (+21)	1 679
17.04.2020	18 105 (+1 959)	127 (+14)	1 517
16.04.2020	16 146 (+1 370)	113 (+7)	1 394
15.04.2020	14 776 (+1 774)	106 (+11)	1 205
14.04.2020	13 002 (+1 489)	95 (+13)	1 016
13.04.2020	11 513 (+1 355)	82 (+10)	837
12.04.2020	10 158 (+1 306)	72 (+14)	687
11.04.2020	8 852 (+1 030)	58 (+8)	499
10.04.2020	7 822 (+1 124)	50 (+12)	350
09.04.2020	6 698 (+857)	38 (+7)	313
08.04.2020	5 841 (+660)	31	270
07.04.2020	5 181 (+697)	31 (+2)	222
06.04.2020	4 484 (+591)	29	206
05.04.2020	3 893 (+536)	29 (+2)	198
04.04.2020	3 357 (+434)	27 (+7)	194
03.04.2020	2 923 (+448)	20 (+1)	168
02.04.2020	2 475 (+595)	19 (+3)	140
01.04.2020	1 880 (+267)	16 (+5)	115
31.03.2020	1 613 (+387)	11 (+5)	70
30.03.2020	1 226 (+212)	6	28
29.03.2020	1 014 (+197)	6 (+2)	28
28.03.2020	817 (+114)	4 (+1)	18
27.03.2020	703 (+157)	3 (+1)	18
26.03.2020	546 (+136)	2	15
25.03.2020	410 (+120)	2 (+2)	15
24.03.2020	290 (+28)	0	9
23.03.2020	262 (+71)	0	9
22.03.2020	191 (+54)	0	8
21.03.2020	137 (+6)	0	8
20.03.2020	131 (+33)	0	5
19.03.2020	98 (+12)	0	5
18.03.2020	86 (+30)	0	1
17.03.2020	56 (+3)	0	1
16.03.2020	53 (+20)	0	1
15.03.2020	33	0	1

14.03.2020	33 *(+9)*	0	1
13.03.2020	24 *(+5)*	0	1
12.03.2020	19 *(+4)*	0	1
11.03.2020	15 *(+6)*	0	1
10.03.2020	9	0	1
09.03.2020	9 *(+3)*	0	1
08.03.2020	6	0	1
07.03.2020	6	0	1
06.03.2020	6 *(+5)*	0	0
05.03.2020	1	0	0
04.03.2020	1	0	0
03.03.2020	1	0	0
02.03.2020	1	0	0
